# Identification of Potential Genes and Critical Pathways in Postoperative Recurrence of Crohn’s Disease by Machine Learning And WGCNA Network Analysis

**DOI:** 10.2174/1389202924666230601122334

**Published:** 2023-10-27

**Authors:** Aruna Rajalingam, Kanagaraj Sekar, Anjali Ganjiwale

**Affiliations:** 1 Department of Life Sciences, Bangalore University, Bangalore, Karnataka, 560056, India;; 2 Laboratory for Structural Biology and Bio-computing, Computational and Data Sciences, Indian Institute of Science, Bangalore, Karnataka, 560012, India

**Keywords:** Crohn's disease, postoperative recurrence, protein-protein interaction (PPI) network, diagnostic biomarker, remission, diagnosis

## Abstract

**Background:**

Crohn's disease (CD) is a chronic idiopathic inflammatory bowel disease affecting the entire gastrointestinal tract from the mouth to the anus. These patients often experience a period of symptomatic relapse and remission. A 20 - 30% symptomatic recurrence rate is reported in the first year after surgery, with a 10% increase each subsequent year. Thus, surgery is done only to relieve symptoms and not for the complete cure of the disease. The determinants and the genetic factors of this disease recurrence are also not well-defined. Therefore, enhanced diagnostic efficiency and prognostic outcome are critical for confronting CD recurrence.

**Methods:**

We analysed ileal mucosa samples collected from neo-terminal ileum six months after surgery (M6=121 samples) from Crohn's disease dataset (GSE186582). The primary aim of this study is to identify the potential genes and critical pathways in post-operative recurrence of Crohn’s disease. We combined the differential gene expression analysis with Recursive feature elimination (RFE), a machine learning approach to get five critical genes for the postoperative recurrence of Crohn's disease. The features (genes) selected by different methods were validated using five binary classifiers for recurrence and remission samples: Logistic Regression (LR), Decision tree classifier (DT), Support Vector Machine (SVM), Random Forest classifier (RF), and K-nearest neighbor (KNN) with 10-fold cross-validation. We also performed weighted gene co-expression network analysis (WGCNA) to select specific modules and feature genes associated with Crohn's disease postoperative recurrence, smoking, and biological sex. Combined with other biological interpretations, including Gene Ontology (GO) analysis, pathway enrichment, and protein-protein interaction (PPI) network analysis, our current study sheds light on the in-depth research of CD diagnosis and prognosis in postoperative recurrence.

**Results:**

PLOD2, ZNF165, BOK, CX3CR1, and ARMCX4, are the important genes identified from the machine learning approach. These genes are reported to be involved in the viral protein interaction with cytokine and cytokine receptors, lysine degradation, and apoptosis. They are also linked with various cellular and molecular functions such as Peptidyl-lysine hydroxylation, Central nervous system maturation, G protein-coupled chemoattractant receptor activity, BCL-2 homology (BH) domain binding, Gliogenesis and negative regulation of mitochondrial depolarization. WGCNA identified a gene co-expression module that was primarily involved in mitochondrial translational elongation, mitochondrial translational termination, mitochondrial translation, mitochondrial respiratory chain complex, mRNA splicing *via* spliceosome pathways, *etc*.; Both the analysis result emphasizes that the mitochondrial depolarization pathway is linked with CD recurrence leading to oxidative stress in promoting inflammation in CD patients.

**Conclusion:**

These key genes serve as the novel diagnostic biomarker for the postoperative recurrence of Crohn’s disease. Thus, among other treatment options present until now, these biomarkers would provide success in both diagnosis and prognosis, aiming for a long-lasting remission to prevent further complications in CD.

## 1 INTRODUCTION

Crohn’s disease (CD) is a chronic inflammatory bowel disease that affects the entire gastrointestinal tract from mouth to anus. It is idiopathic with unknown etiology [1, 2]. The incidence of CD annually ranges from 3 to 20 cases per 100,000 population [3], whose prevalence and incidence are more significant and common in industrialized economies and is reported to steadily increase in countries like Asia [4, 5]. It is characterized by discontinuous skip lesions, whose inflammation is classically transmural, affecting any segment of the gastrointestinal tract [6]. This is not a sex-specific disease and is found to be equally distributed in males and females whose symptoms are variable but primarily presented as diarrhea, abdominal pain, weight loss, nausea, vomiting, and sometimes fevers or chills. Interactions between various genetic, environmental, intestinal microflora, infectious, immunological, and dietary factors result in abnormal mucosal immune response making its pathophysiology complicated to understand [7]. Currently, there is no cure for this disease, but seeking the proper therapeutic approach is essential to assess disease severity and prognostic factors resulting in complications [7].

Strategies for treating CD should be aimed at a deep and long-lasting remission to prevent further complications of developing the disease. But most patients opt for surgery to control their symptoms and improve their quality of life. It is reported that nearly 80% of CD patients require surgery during their lifetime [8]. Between 1970 and 1988, 1379 patients with Crohn's disease were treated at the University of Chicago, out of which 639 required at least one surgical procedure to improve their quality of life. Among these 639, 317 were women, and 322 were men. Their mean age is reported as 32.5 years. Common indications of these patients undergoing surgery include 215 patients, *i.e*., 33% were not responding to any medical treatments, 154, *i.e*., 24% were suffering from fistula, and 141, *i.e*., 22% had a bowel obstruction. Of the 582 patients, 416 had primary anastomosis, 124 had temporary stroma, and 42 had permanent stroma. The remaining 57 patients underwent stricturoplasty, bypass, and other diverse procedures. Two patients died, and 118 had recurrence during reoperation. Thus, surgical treatments are not curative; most patients will eventually suffer a recurrence within weeks to months of surgery [9]. So, the rate of postoperative recurrence (P.O.R.) is high, reporting a rate of 35 - 85% after their first year of surgery, and by the third year, it increases to 85 - 100% cases, with 34 - 86% of patients being symptomatic. So, a 50 - 60% reoperation rate is generally reported [10]. Thus, most CD patients often experience a period of frequent recurrence after their surgery.

Several risk factors have been studied that increase the risk of future recurrences. Age at onset of disease, sex, family history of Crohn’s disease, smoking, duration of Crohn’s disease before surgery, prophylactic medical treatment (corticosteroids, 5-amino salicylic acid [5-ASA], and immunosuppressants), anatomical site of involvement, indication for surgery (perforating or non-perforating disease), length of resected bowel, anastomotic technique, presence of granuloma in the specimen, involvement of infection at the resection margin, blood transfusions, and postoperative complications influence the recurrence rate after surgery [11-20].

There are few studies on understanding the genetics or genetic interactions of postoperative recurrence in CD. Various studies on CD continuously implicate NOD2 as an important susceptibility gene in CD pathogenesis, among which the Nod2 1007fs (Nod2fs) frameshift mutation is the most prevalent mutation [21, 22]. However, a study on meta-analysis concluded there is no sufficient data to support NOD2 in predicting disease recurrence, but they did not exclude its importance for CD [23]. Fowler and his colleagues in the year 2014 reported a novel association between SMAD3 and increased risk of repeat operation and shorter time to repeat surgery [24]. Later in 2018, BACH2 was reported as a susceptibility locus for postoperative recurrence of Crohn’s disease and was found to be critical in the differentiation and function of T cells. It is also associated with several dysregulated immunologic phenomena [25]. CARD8 gene expression is high on both monocytes and gut epithelium and is also independently associated with surgical recurrence in CD [26]. In 2015, Germain and his colleagues also confirmed the CARD8 gene variant as a risk factor for postoperative recurrence of Crohn’s disease [27]. Very little data is available on genetic factors contributing to postoperative recurrence and the molecular mechanism involved.

Microarray technology identifies millions of gene expression profiles of millions of genes. It is a high-throughput, robust, and widely used approach. Gene Expression Omnibus (GEO) comprises important information on microarray technology, through which public data could be integrated and re-analyzed, providing valuable information and novel perspectives regarding different diseases. Weighted gene coexpression network analysis (WGCNA) is a well-known method for studying biological networks based on pairwise correlations between variables. This is one of the prominent approaches in data mining. It gives a weighted gene coexpression network and helps in the detection of highly correlated gene modules, the association of gene modules with clinical traits, and the identification of intra-modular hub genes [28]. WGCNA is widely used in bioinformatics analysis of many diseases, including CD. For instance, Gene Ontology (GO) enrichment of modules defined by WGCNA showed several immune response-related genes that play potential roles in CD [29].

Our study attempts to find the hub genes, genetic interactions, and gene-trait relationship of Crohn’s disease’s postoperative recurrence to understand its pathogenesis better, aiming for a long-lasting remission to prevent further complications. The GSE186582 dataset represents studies on Ileal samples with Crohn’s disease. In the present study, we have coupled machine learning with DEG analysis to identify significant gene markers contributing to the pathophysiology of postoperative recurrence of Crohn’s disease. As a branch of artificial intelligence, machine learning has emerged as a valuable tool in clinical research. However, the prediction accuracy of the machine learning model is inversely proportional to a number of features, so selecting features for the model should be as appropriate, limited, and informative as possible [30]. WGCNA, in conjunction with DEG, is employed to correlate each module with different clinical traits like smoking, biological sex (males and females), *etc*., associated with CD disease recurrence. Data analysis by this approach results in selecting genes involved in the pathogenesis of postoperative recurrence. The significance of the selected genes was confirmed to distinguish between remission and recurrence samples which derive insightful inferences on biological functions and pathways involved in the postoperative recurrence of Crohn’s disease.

## 2 MATERIALS AND METHODS

### 2.1 Data Collection and Gene Expression Analysis

#### 2.1.1 Data Collection and Pre-processing

The gene expression profile of Ileal samples for Crohn’s disease was downloaded from the Gene Expression Omnibus database (GEO, https://www.ncbi.nlm.nih.gov/geo/)) with *Homo sapiens* as the inclusion criteria. GSE186582 reports microarray analysis of mRNA isolated from Ileal samples comprising Crohn’s disease patients and healthy samples from non-inflammatory controls. The dataset was detected using Affymetrix Human Genome U133 Plus 2.0 Array [HG-U133_Plus_^2^] containing four hundred and eighty-nine samples (489) with inflamed ileum (M0I) and the ileal margin (M0M) at the time of surgery and during postoperative endoscopy six months later (M6) comprising 326 recurrence and 138 remission samples. Twenty-five ileal non-IBD control biopsies (Ctrl) from patients who underwent ileocecal resection for colonic tumor with healthy ileum represent control samples. The current study focussed only on M6 neoterminal ileum samples, out of which 121 samples (37 Remissions and 84 Recurring) were analyzed to observe any variation in gene expression patterns between recurring and remission samples.

Affymetrix CEL files were downloaded from NCBI GEO accession number GSE186582 and re-normalized using the (Robust Multiple Array Average) RMA method in AltAnalyze software [31, 32]. Outliers in the datasets were detected and removed using the interquartile range (Supplementary Information **A1**).

#### 2.1.2 Differentially Expressed Genes (DEG) Analysis

GEO2R analysis tool with the limma package from Bioconductor to display the t-test score, p-test score, and adjusted p-score was used to analyze differentially expressed genes of the CD dataset (https://www.ncbi.nlm.nih.gov/geo/geo2r/). The top 250 DEGs from the dataset were obtained by setting forced normalization and the significance level cut-off (*p* ≤0.05), and their values were adjusted using Benjamini-Hochberg correction (false discovery rate) (Supplementary Information **A2**) [33]. The analysis was run with the default parameters in the GEO2R tool to obtain the top 250 DEGs for the dataset for further analysis. The data was visualized using heatmaps. The data structure in the set of 2-fold differentially expressed genes was analyzed using the complete-linkage (farthest neighbor) algorithm with the ‘Euclidean metric measure.

### 2.2 Weighted Gene Co-expression Network Analysis (WGCNA) Analysis

Python package WGCNA (PyWGCNA) was used to construct a coexpression network in line with the WGCNA flow. This was first described by Langfelder and Horvath using the R package [28]. PyWGCNA (Version 1.15.0) is used to construct the coexpression network of DEGs. First, we used the TPM matrix to create a PyWGCNA object. This object serves as a container that contains expression data and analysis like clustering and visualizing results for DEGs. These were stored as an anndata in GeneExp class. Second, the samples were clustered to identify any obvious outliers. This was achieved using preprocess function to remove genes with too many missing values or low expressed genes across samples. Third, the coexpression network was constructed. The soft thresholding power β, to which coexpression similarity was raised to calculate adjacency, was calculated; clustering using Topological Overlap Matrix (TOM) was achieved, and merging of modules whose expression profiles are very similar was constructed using the findModules function. Fourth, key gene modules were identified using hierarchical clustering and the dynamic tree-cut function. Fifth, after identifying modules, it is further analyzed using the analyse WGCNA function to quantify the module-trait relationship and gene-ontology analysis to determine the important key genes associated with postoperative recurrence.

After the modules were identified, the module eigengenes (ME) was summarized by the first principal component of the module expression levels. Module–trait relationships were estimated using the correlation between MEs and traits, allowing efficient identification of the relevant modules. To evaluate the correlation strength, we calculated the module significance (MS), defined as the average absolute gene significance (GS) of all the genes in the module. The GS was measured as the log10 transformation of the *P*-value (logP) in the linear regression between gene expression and traits. In the WGCNA, modules with the highest MS score are usually defined as the key module and selected for further analysis.

### 2.3 Machine Learning Binary Classifiers

Logistic Regression (LR), Decision tree classifier (DT), Support Vector Machine (SVM), Random Forest classifier (RF), and K-nearest neighbor (KNN) classifier [34] were used as binary classifiers between remission and recurrence samples. The data was divided into 20% test and 80% train using the test-train split() function from scikit learn python library with 10 fold cross validation. The dataset was scaled with MinMax Scalar and Power Transformed to obtain the normal Gaussian distribution. All five algorithms were implemented using the Scikit-learn machine learning library (Version 0.21.2) [35]. Feature selection for marker genes was performed using Recursive feature elimination (RFE) and Random Forest classifier (RFC) [36, 37] using the Scikit-learn python module [35, 38].

### 2.4 Identification and Validation of Marker Genes

The genes selected by RFE and RFC were validated using LR, DT, SVM, RF, and KNN with test-train split () and 10-fold cross-validation as used in binary classifiers between remission and recurrence samples. The performance of all five classifiers was measured using accuracy, precision, and area under the ROC curve (AUC) as the performance metrics [39-41].

### 2.5 Functional Enrichment Analysis

To explore the enriched biological pathways and annotations of selected marker genes in terms of Gene Ontology (GO) and the Kyoto Encyclopedia of Genes and Genomes (KEGG), Database for Annotation, Visualization and Integrated Discovery (DAVID) v6.8 (https://david.abcc.ncifcrf.gov) [42] and a graphical tool for gene set enrichment analysis - ShinyGO (Version 0.76) (http://bioinformatics.sdstate.edu/go/) was used to conduct the Functional, Biological, and Cellular GO Categories. The enriched pathways were defined based on the *p*-value cut-off (false discovery rate (FDR)) below 0.05 [43]. To understand the biological meaning of the DEGs and modules obtained from WGCNA, GSEApy (Python package) was used for performing GO enrichment and KEGG pathway analyses. All the results were validated with a statistical significance of *p*-value < 0.05. Gene-Gene interaction network was performed for the selected biomarker genes through the GeneMANIA Cytoscape plugin command-line tool [44].

### 2.6 Chemical - Gene Interaction Network Analysis

To provide detailed information such as chemical gene/protein interactions, chemical diseases, and gene-disease relationships, the comparative toxicological genomics database (CTD, http://ctdbase.org/) was used [45]. Diagnostic markers and the molecular compound interaction network were visualized using Cytoscape software.

## 3 RESULTS

### 3.1 Data Preparation and Preliminary Data Analysis

The raw data CEL files of GSE186582 (326 recurrences, 138 remissions, and 25 control samples) consist of 196 samples that are of inflamed ileum (M0I), 147 of ileal margin (M0M) at the time of surgery, and 121 obtained during postoperative endoscopy six months later (M6) and 25 ileal non-IBD control biopsies (Ctrl) from patients who underwent ileocecal resection for colonic tumor with healthy ileum represent control samples.

Preliminary data analysis of the GSE186582 dataset showed an equal number of males and females in distribution. It has both smokers and non-smokers with a more significant number of recurrence samples in inflamed ileum (M0I) compared to other samples (M0M, M6) (Figs. **S1A-D**). Analysis based on smoking infers that smoking increases the risk of recurrence in males compared to females in recurring groups. In remission or non-recurring groups, smoking does not bring any changes in both males and females. Data analysis on patients who undergo postoperative anti-TNF treatment infers that smoking is associated with the risk of recurrence and reduces the chance of remission in non-recurring groups (Figs. **S2A-B**).

Out of these, only M6 samples (37 Remissions and 84 Recurring) were taken into analysis. M6 data was processed using AltAnalyze v.2.1.4.3 (http://www.altanalyze.org) after downloading it from the Gene Expression Omnibus database (GEO, https://www.ncbi.nlm.nih.gov/geo/). The RMA method (Robust Multiple Array Average) was applied to all Affymetrix CEL files, detection above background (DABG) with *p*-value < 0.05, and the probe sets were annotated. All the GSM files were grouped as ‘Rec’ for recurrence samples and ‘Rem’ for remission samples. Data with missing ‘Gene Symbols’ was dropped, and a total of 16,384 gene features were selected for further analysis. Outlier detection and treatment in the dataset were performed using the interquartile range (IQR) (Supplemental Information **A1**).

### 3.2 DEG Analysis

**Fig. (1):**
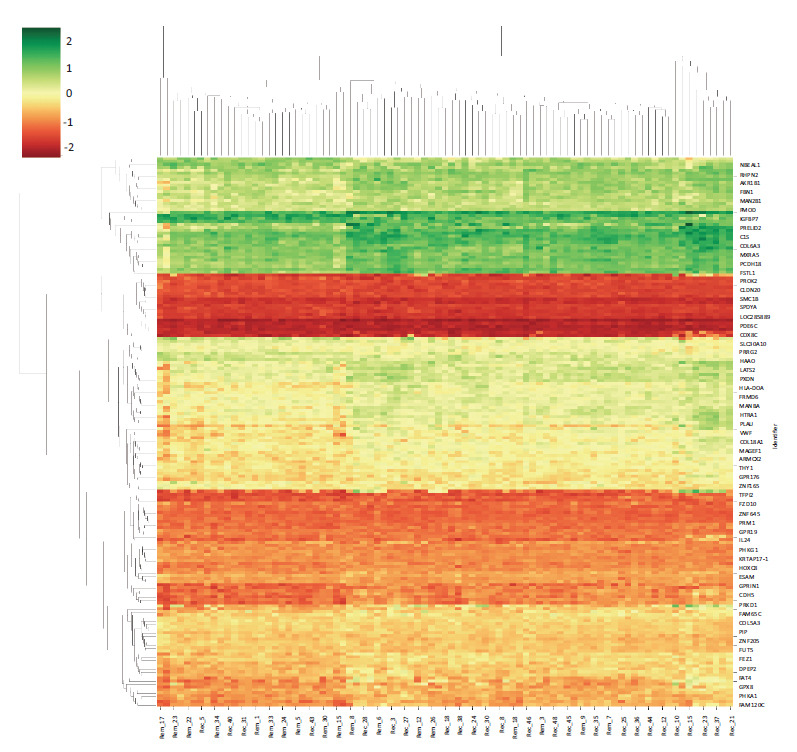
Differential gene expression analysis represented for GSE186582 heatmap and cluster map.

The GEO2R analysis tool was used to find the top DEGs between remission and recurring samples. The top 250 differentially expressed genes across the dataset were analyzed using the default parameters inbuilt into the GEO2R tool by applying forced normalization, log transformation, and an adjusted *p*-value of < 0.05. A total of 250 DEGs were identified from the analysis (Supplemental Information **A2**). Obtained GEO2R results were merged with Affymetrix CEL files (Supplemental Information **A3**), resulting in a total of 183 gene features to plot heatmap for differentially regulated genes between remission and recurrence samples to observe if there is any distinction between them (Fig. **1**). Obtained 183 gene features were taken for functional enrichment analysis.

### 3.3 Weighted Gene Co-expression Network Analysis (WGCNA) Analysis

**Fig. (2):**
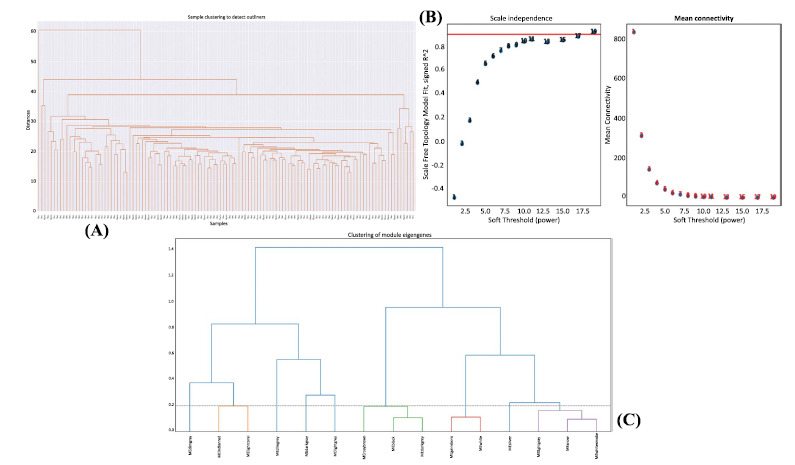
WGCNA-based identification of coexpression modules of GSE186582 dataset. (**A**) The clustering dendrogram of M6 samples with tree leaves corresponding to individual samples detecting any outliers. (**B**) The scale-free fit index for Soft thresholding powers (β) and scale-free topology fitting indices (R2).The soft-thresholding power in the WGCNA was determined based on a scale-free R2 (R2=0.85). The left panel shows the scale-free fit index (y-axis) as a function of the soft-thresholding power (x-axis). The right panel displays the mean connectivity (degree, y-axis) as a function of the soft-thresholding power (x-axis). To maximize model fit, a β=17 was chosen. (**C**) Clustering of module eigengene shows interaction relationships of 15 modules and the hierarchical clustering tree was produced.

**Fig. (3):**
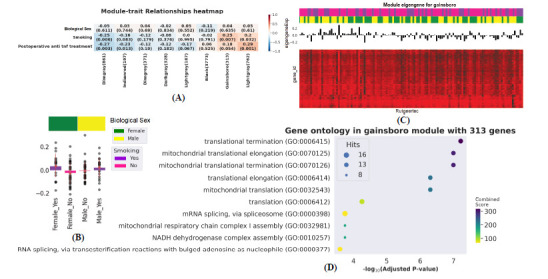
WGCNA analysis identified the gene modules related to postoperative recurrence of CD. (**A**). The heatmap of module trait relationships for CRD (Crohn’s recurrence disease) showed the correlation between each gene module and the clinical status of samples. Each row corresponds to a module eigengene and each column to a trait. Each cell contains the corresponding correlation and p-value. The table is color-coded by correlation according to the color legend, which decreased in size from red to blue. The red cube represented a positive correlation, whereas the blue cube represented a negative correlation. The consensus correlation between modules and phenotypes was reported as a number shown in each cube, with *p*-value (in parenthesis) printed below the correlations. The Gainsboro module shows a good positive correlation with Smoking status. (**B**) Module Eigengene for gainsboro showed smoking increases the chance of recurrence both in males and females. (**C**) Heatmap of Module Eigengene for gainsboro showed the overall expression of eigengenes in this module. Pink and violet colour legend represents non-smokers and smokers status with yellow and green legends showing the distribution of male and female in the heatmap with respect to selected module. (**D**) GO analyses of the gainsboro module (Functional Enrichment Analysis).

Correlated genes usually exhibit identical or similar expression patterns. To gain a deeper insight into gene correlation in postoperative recurrence, we established a co-expression network to screen gene modules that contain highly correlated genes using WGCNA. Next, we analyzed network topology for threshold powers from 1 to 19 and identified the relatively balanced scale independence and mean connectivity of the WGCNA. As the lowest power for the scale-free topology fit index of 0.85, power value 19 was selected to produce a hierarchical clustering tree (Figs. **2A** and **B**). Then we used the WGCNA to assign genes with similar expression patterns that resulted in 15 gene modules. Interaction relationships of 15 modules were analyzed, and the hierarchical clustering tree was produced (Fig. **2C**). The results revealed that eight modules were independent of each other, suggesting a good level of independence of gene expression in each of the eight modules. WGCNA was then used to correlate each module with three different clinical traits, such as biological sex, smoking, and postoperative anti-TNF treatment, by calculating MS for each module–trait correlation (Fig. **3A**).

Fig. **3A** shows up to eight modules representing clusters with highly correlated genes (Dimgray, Indianred, Dimgrey, Darkgray, Lightgrey, Black, Gainsboro, and Lightgray). The color of the module indicates the degree of correlation from a strong positive correlation red to a strong negative correlation blue. The postoperative anti-TNF treatment showed a negative correlation for five modules (Dimgray, Indianred, Dimgrey, Darkgray, Lightgrey) and a positive correlation for three modules (Black, Gainsboro, and Lightgray). Biological sex showed a positive correlation for five modules (Indianred, Dimgrey, Lightgrey, Gainsboro, and Lightgray), while other modules showed a negative correlation.

Further analysis on each module resulted in a heatmap of 8 module eigengenes. This showed the overall expression of module eigengenes (Figs. **S3A-H**) that relates to the four clinical traits. The Gainsboro module showed a slight positive correlation with smoking than other modules (r = 0.25 and *P* = 0.007). Further analysis of the gainsboro module containing 313 genes associated with smoking increases the chance or rate of recurrence both in males and females (Fig. **3B**). Heatmap of Module Eigengene for gainsboro showed the overall expression of eigengenes in this module (Fig. **3C**). In contrast, five modules (Dimgray, Indianred, Dimgrey, Darkgray, and Black) negatively correlate with smoking (Fig. **3A**). Figs. **S4A-H** plots show the correlation of each module and its association with smoking concerning both sex distribution, among which the Gainsboro module shows good correlation than other modules.

Genes in these eight modules were subsequently used for Gene ontology (GO) enrichment analysis. The enrichment analysis results of 8 modules are shown in Figs. **S5A-H**. The Dimgray module contains a total of 861 genes. These genes were significantly enriched in protein ubiquitination, cytosolic transport, protein modification by small protein conjugation, mRNA splicing, *etc*. (Fig. **S5A**). The Indianred module contains a total of 197 genes significantly enriched in the regulation of transcription RNA polymerase II, RNA splicing, negative regulation of cellular macromolecule biosynthetic process, and so on (Fig. **S5B**). For Dimgrey module contains a total of 371 genes that are significantly enriched in the cytokine-mediated signaling pathway, positive regulation of T cell activation, neutrophil degranulation, and neutrophil activation in immune response, and so on (Fig. **S5C**). The Darkgray module, it contains a total of 328 genes. These genes are significantly enriched in mitochondrial translational elongation, translational termination, mitochondrial translational, translational termination, and so on (Fig. **S5D**). The Lightgrey module contains a total of 187 genes. These genes were significantly enriched in mitotic spindle organization, microtubule cytoskeleton organization involved in mitosis, DNA metabolic process, and so on (Fig. **S5E**). The Black module contains a total of 3773 genes significantly enriched in positive regulation of transcription, regulation of the apoptotic process, positive regulation of transcription by RNA polymerase II, and so on (Fig. **S5F**). The Gainsboro module, it contains a total of 313 genes. These genes are significantly enriched in translational termination, mitochondrial translational elongation, mitochondrial translational termination, and mRNA splicing (Figs. **3D** and **S5G**). The Lightgray module contains a total of 762 genes that were significantly enriched in neutrophil degranulation, neutrophil activation in immune response, neutrophil-mediated immunity, intracellular protein transport so on (Fig. **S5H**).

### 3.4 Machine Learning-based Selection of Marker Genes

**Fig. (4):**
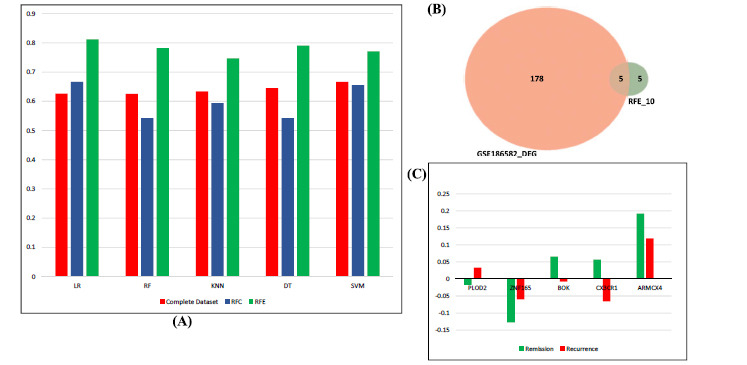
(**A**) Classification accuracy with feature selection. Recursive feature elimination (RFE) shows an increase in training accuracy as compared to random forest classifier (RFC). Abbreviations: LR: Logistic Regression, RF: Random Forest, KNN: K-Nearest Neighbour, DT: Decision Tree, SVM: Support Vector Machine (**B**) Common gene features selected with GSE186582_DEG and RFE (**C**) Gene regulation of 5 gene features are compared to Remission samples.

The GSE186582 dataset with 19655 gene features was used for machine learning based selection of marker genes. The ‘Rem’ sample was encoded to ‘0’, and the ‘Rec’ sample was encoded to ‘1’ to convert the categorical features to the numeric features. Binary classification algorithms with gene feature as predictor variables were used to classify Remission’ 0’ *vs.* Recurrence’ 1’ as target variables. The comparative performance of five different classifier algorithms, Logistic Regression (LR), Decision tree classifier (DT), Support Vector Machine (SVM), Random Forest classifier (RF), and K-nearest neighbor (KNN) classifiers, is as shown in Fig. **4A**. Classification without feature selection (Figs. **S6A** and **S6B**) showed significantly less accuracy compared to the two different feature selection algorithms, Recursive Feature Elimination (RFE) and Random Forest classifier (RFC). The bias in the models was reduced by applying a 10-fold cross-validation approach. Logistic regression showed an accuracy of 81.22% and AUC_ROC of 0.91, KNN with 74.77% accuracy and AUC_ROC of 0.82, DT with 79% accuracy and AUC_ROC of 0.76, SVM with 77.11% accuracy and AUC_ROC of 0.90 and RF with 78.22% accuracy and AUC_ROC of 0.89. Dimensionality reduction and selection of important features were performed using two different feature selection algorithms, Recursive Feature Elimination (RFE) and Random Forest classifier (RFC). RFE can be implemented using two different configuration options: the number of selected features and the classification algorithm choice. RFE selected this study’s top 10 gene features with LR as the classifier (Figs. **S7A-C**). Since RFE works by removing the features step by step and fitting the model on the training data until the desired number remains, it becomes computationally expensive. Random Forests are tree-based strategies that rank the features by reducing the mean ‘Gini impurity’ on the overall trees. The top 15 features were selected by RFC (Figs. **S8A-C**). The selected features by RFE and RFC were used to check the classification performance by retraining the selected features (Fig. **4A**). Features selected by RFE improved the accuracy and AUC of all the five classifiers; in contrast, RFC reduced the accuracy and AUC considerably. The differential gene expression analysis of 10 gene features selected by RFE is represented by a cluster map (Fig. **S8C**). The key genes were selected by selecting the common genes obtained from the intersection of DEG (Rem *vs* Rec) and RFE (Fig. **4B**). Our study could identify 5 ‘Biomarkers’ for postoperative recurrence. Thus, ‘PLOD2’ (Procollagen-Lysine,2-Oxoglutarate 5-Dioxygenase 2), ‘ZNF165’ (Zinc Finger Protein 165), ‘BOK’ (Bcl-2 related ovarian killer), ‘CX3CR1’ (C-X3-C Motif Chemokine Receptor 1), and ‘ARMCX4’ (Armadillo Repeat Containing X-Linked 4) are the novel marker genes identified in the study. Four biomarkers identified are downregulated except PLOD2 (Upregulated) compared with remission samples (Fig. **4C**).

**Fig. (5):**
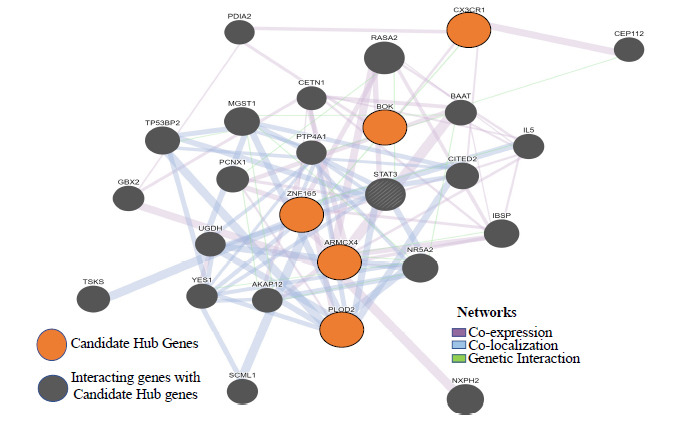
GeneMANIA Cytoscape composite network of PLOD2, ZNF165, BOK, CX3CR1 and ARMCX4 showing 20 related genes, with 26 total genes and 94 total links.

The gene-gene interaction network predicted for proposed biomarker genes through the GeneMANIA Cytoscape plugin command-line tool is shown in (Fig. **5**). The network shows 20 related genes, with 26 total genes and 94 total links based on protein-protein interaction data collected from BioGRID and PathwayCommons, genetic interaction data, shared protein domains, co-localization, pathway data, and predicted functional relationships between the genes [44]. The network shows PLOD2, ZNF165, and ARMCX4 co-expressed with STAT3 (Signal transducers and transcription activators of transcription 3) and IL-5 (interleukin-5). Studies on STAT3 report its involvement in various autoimmune disorders, including inflammatory bowel disease (IBD), in which its activation in acquired immunity results in a pathogenic role in colitis, whereas its activation in innate immunity results in a suppression of colitis [46]. It has been reported that STAT3 phosphorylation at the surgical ileal margin was associated with severe recurrence in Crohn’s disease patients [47]. Also, its persistent activation results in the activation of many inflammatory pathways, such as nuclear factor-κB (NF-κB) and interleukin-6 (IL-6)–GP130–Janus kinase (JAK) pathways. Thus, STAT3 has a leading role in cancer, inflammation, and immunity [48]. On the other hand, IL-5 and IL-6 share a common protein domain in which IL-5 activation results in the activation of B-cells and eosinophils regulating innate and acquired immune responses [49]. PLOD2, STAT3, ZNF165, TP53BP2, CITED2, IL-5, and YES1 are found to be co-localized in the network. Tumor suppressor p53-binding protein 2 (TP53BP2) suppresses the tumorigenesis process. It also helps in the regulation of proliferation, apoptosis, autophagy, and migration. It activates many signaling pathways such as NF-κB, RAS/MAPK (RAS-mitogen activated protein kinase), mevalonate, TGF-β1 (Transforming growth factor beta 1), PI3K/AKT (phosphoinositide 3-kinase (PI3K)/protein kinase B (AKT) pathway) and autophagy pathways [50]. CITED2 (Cbp/P300 Interacting Transactivator With Glu/Asp Rich Carboxy-Terminal Domain 2) is an intrinsic negative regulator of inflammation whose deficiency enhances inflammatory response gene expression in macrophages, thereby activating pro-inflammatory response at the site of inflammation [51]. YES1 (YES Proto-Oncogene 1, Src Family Tyrosine Kinase) is a nonreceptor protein tyrosine kinase involved in regulating cell growth and survival, apoptosis, cell-cell adhesion, cytoskeleton remodeling, and differentiation. Recent findings suggest that YES1 can be a therapeutic target in cancer [52]. Thus, most of the cancer-related genes are co-localized with biomarker genes. The network also shows genetic interaction between CX3CR1, IL-5 along with ZNF165, and STAT3 with RASA2. RASA2 (RAS P21 Protein Activator 2) is a member of the GAP1 family of GTPase-activating proteins. The gene product stimulates the GTPase activity of normal RAS p21 but not its oncogenic counterpart. Acting as a suppressor of RAS function, the protein enhances the weak intrinsic GTPase activity of RAS proteins resulting in the inactive GDP-bound form of RAS, thereby allowing control of cellular proliferation and differentiation. Thus, it acts as a negative regulator of RAS, whose mutation results in melanoma [53]. Therefore, the interacted genes are involved in inflammation, cancer, and immunity (Supplementary Information **A4**).

### 3.5 Functional Enrichment Analysis

**Fig. (6):**
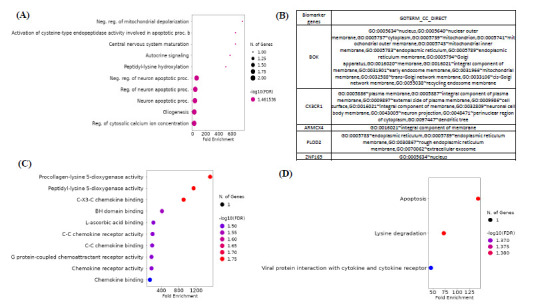
Functional enrichment analysis of biomarker genes. (**A**) Biological process (**B**) Cellular components (**C**) Molecular functions (**D**) KEGG pathway. Top pathways are sorted by fold enrichment enriched with significant DEGs.The dot size represents the count of differentially expressed genes, and the color depth represents the enrichment FDR. Detailed functional enrichment analysis has been provided in the supplemental information.

Functional enrichment analysis showed the importance of biomarker genes in various significant pathways and biological components. In Biological processes (BP), the genes were enriched in negative regulation of mitochondrial depolarization, peptidyl-lysine hydroxylation, cytosolic calcium ion concentration regulation, gliogenesis, and activation of cysteine-type endopeptidase activity involved in apoptotic process b and so on (Fig. **6A** and Supplementary Information **A5**). In the Cellular component (CC), the genes were enriched in the nucleus, nuclear outer membrane, mitochondrion, mitochondrial outer and inner membrane, endoplasmic reticulum membrane, and so on (Fig. **6B** and Supplementary Information **A6**). In Molecular Functions (MF), the genes were enriched in G-protein coupled receptor activity, chemokine receptor activity, BH domain binding, Procollagen-lysine 5-dioxygenase activity, peptidyl-lysine 5-dioxygenase activity, L-ascorbic acid binding, C-X3-C chemokine binding, and so on (Fig. **6C** and Supplementary Information **A7**). The KEGG enrichment analysis showed that these marker genes involve Apoptosis, Lysine degradation, and viral protein interaction with cytokine and cytokine receptors (Fig. **6D** and Supplementary Information **A8**). Figs. (**S9** and **S10**) show a detailed picture of the involvement of the marker genes in Lysine degradation and viral protein interaction with cytokine and cytokine receptors. Thus, these pathways are mainly involved in immune cell activation, innate immunity, and acquired immunity for the host’s defense to maintain a healthy immune system.

### 3.6 Chemical - Gene Interaction Network Analysis

**Fig. (7):**
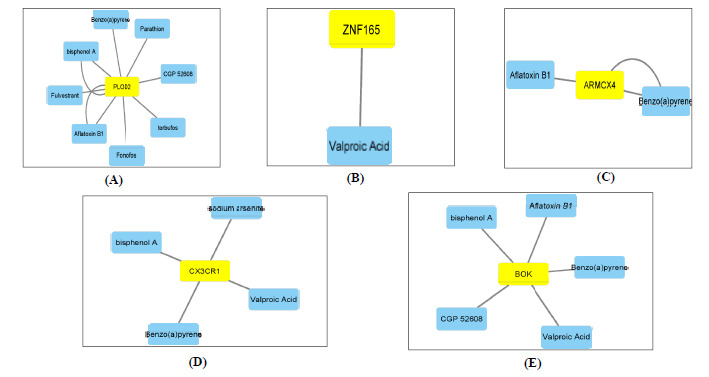
Chemical-gene interactions network with therapeutic drugs and chemical compounds and five diagnostic genes was constructed using the CTD database. (**A-E**). The interaction between existing therapeutic drugs and the diagnostic genes. (**A**) PLOD2. (**B**) ZNF165. (**C**) ARMCX4. (**D**) CX3CR1. (**E**) BOK.

The chemical or therapeutic compounds interacting with the diagnostic genes PLOD2, ZNF165, BOK, CX3CR1, and ARMCX4 were searched in the CTD database. The interaction network was created and visualized using Cytoscape (Fig. **7**). The network showed that multiple chemicals could affect the expression of these five diagnostic genes. PLOD2 shows interaction with several chemicals such as Bisphenol A, Benzo(a)pyrene, Fulvestrant, *etc*., in which all of these chemicals results in increased or decreased methylation of PLOD2 genes affecting its. ZNF 165 shows its interaction with valproic acid, which helps in the decreased methylation of ZNF 165 expression. ARMCX4 interaction with Benzo(a)pyrene and Aflatoxin B1 affects the methylation of ARMCX4 intron and promotor [55, 57]. CX3CR1 interaction with Bisphenol A results in decreased methylation of CX3CR1 gene. Its interaction with Benzo(a)pyrene affects the methylation of CX3CR1 exon, valproic acid and sodium arsenite interaction increases the methylation of CX3CR1 gene expression [54, 56-58]. On the other hand, BOK gene had interaction with CGP 52608, Bisphenol A, Benzo(a)pyrene, Aflatoxin B1 and valproic acid which alters the methylation of BOK introns, promoter and gene [54-57, 59] (Supplementary Information **A9**).

## 4 DISCUSSION

Crohn's disease (CD) is a chronic inflammatory bowel disease that affects the entire gastrointestinal tract from mouth to anus [1, 2]. Currently, this disease has no cure, but present treatment options aim to control the inflammation and symptoms. But most patients who suffer from complications primarily opt for surgical treatment to improve their quality of life. However, surgical treatments are not curative; most patients suffer a recurrence within weeks to months of surgery [10]. Thus, it is essential to understand postoperative recurrence genetics or genetic interactions for a deep and long-lasting remission. Present advances in our understanding of the human genome, gene expression, and genetic architecture are making a path for new opportunities to understand the root cause of this disease and advance the vision of developing improved strategies for diagnosing and prognosis of CD. But there is not much data on the genetics of postoperative recurrence. So, our study attempts to find hub genes, genetic interactions, and gene-trait relationship of postoperative recurrence of Crohn's disease to understand its pathogenesis better to obtain a more accurate diagnosis for long-lasting remission. We conducted a transcriptomic analysis for the GSE186582 dataset, which was retrieved from the GEO database, representing Ileal samples with Crohn's disease. A comparison of the differentially expressed genes from the selected dataset and RFE, the machine learning method identified five key genes that are involved in viral protein interaction with cytokine and cytokine receptors, lysine degradation, apoptosis, Collagen metabolism, Peptidyl-lysine hydroxylation, G protein-coupled chemoattractant receptor activity, BCL-2 homology (BH) domain binding, negative regulation of mitochondrial depolarization and so on. On the other hand, WGCNA analysis confirmed that smoking (from module-trait analysis) increases the risk of postoperative recurrence of Crohn's disease both in males and females. This analysis identifies a gene co-expression module (gainsborro module) that had a good correlation with smoking compared to other modules in which it contains a total of 313 genes that were primarily involved in mitochondrial translational elongation, mitochondrial translational termination, mitochondrial translation, mitochondrial respiratory chain complex, mRNA splicing *via* spliceosome pathways, *etc*., All of these pathways are linked to mitochondrial functions and cellular processes. Any defect in these processes will exacerbate mitochondrial depolarization and reduce cell viability. One of the identified biomarker genes, BOK is also involved in mitochondrial depolarization, in which studies report that this gene promotes mitochondrial fission in preeclampsia. Mitochondrial fission is due to an imbalance in mitochondrial oxidative stress that results in mitochondrial fragmentation, contributing to mitochondrial and cell dysfunction [60, 61]. Studies have reported that postoperative recurrence of Crohn’s disease is associated with the upregulation of mitochondrial dysfunction due to increased activation of the JAK/STAT pathway at the time of surgery [47]. The pathways (Lysine degradation, apoptosis, and viral protein interaction with cytokine and cytokine receptor) identified in this study have also been reported in ulcerative colitis, strengthening the significance of the data identified [62-64]. The gene-gene interaction network of the biomarker gene indicates the involvement of STAT3 and IL-5 genes linked to Crohn's disease's postoperative recurrence. Apart from gene-gene interactions, certain environmental factors, such as tobacco use, are also linked with multiple genes causing Crohn's disease [65, 66].

Histological examination of the Ileal margin and Ileal tissue shows a complete absence of any inflammation. Still, the analysis at the molecular level reveals an inflammatory state at the margin, which could be due to the increased phosphorylated STAT3 protein level at the margin causing T cell clonal expansion associated with disease recurrence [67]. Our study also showed three biomarkers (PLOD2, ZNF165, and ARMCX4) that are co-expressed with STAT3 and IL-5 in the gene-gene interaction network (Fig. **5**). Activation of these genes at the ileal margin results in the activation of many inflammatory pathways especially JAK/STAT signaling pathways that are associated with postoperative recurrence of CD [68].

The interaction network constructed in this study and results obtained from the module-trait relationship from WGCNA analysis shows STAT3 may be linked with smoking that doubling the risk of postoperative recurrence in both males and females, as reported by Li *et al*. in the year 2016 [66].

In the present study, we have coupled machine learning with DEG analysis to identify marker genes contributing to the pathophysiology of postoperative recurrence of Crohn’s disease. A total of 5 biomarker genes, such as PLOD2, ZNF165, BOK, CX3CR1, and ARMCX4, were identified to be common in both RFE-based and DEG-based gene feature selection in the study. Out of 5, 4 biomarkers are downregulated except PLOD2 (Upregulated) compared to remission samples (Fig. **4C**).

PLOD2 (Procollagen-Lysine,2-Oxoglutarate 5-Dioxy- genase 2) gene encodes for a membrane-bound homodimeric enzyme that facilitates by catalyzing the hydroxylation of lysyl residues in collagen-like peptides. This is very important to maintain the stability of intermolecular crosslinks. Thus, the fibril-forming collagens are the most abundant in vertebrates, providing tissue form and stability [69]. Excessive accumulation of collagen results in fibrosis, in which intestinal fibrosis is one of the most common complications in a CD that result in stricture formation in the small intestine and colon, due to which most CD patients opt for surgery [70]. Van Haaften and his colleagues in the year 2020 reported that fibrotic terminal ileum obtained from CD patients show high expression of collagen metabolism genes. They also have observed enzymes such as lysyl hydroxylases 1-3 (PLOD1, PLOD2, and PLOD3), prolyl 4-hydroxylases, prolyl-3-hydroxylases 1-3 expression was more in fibrosis and fibrotic CD compared to non-fibrotic CD patients [71]. Van der Slot and his colleagues also confirmed that the PLOD2 gene is an important enzyme linked with fibrosis [72]. Moreover, abnormal lysine degradation is also linked to the progression of collagen-related diseases and cancer [73, 74]. It is overexpressed in various cancer, especially cervical cancer (CD is a risk factor for cervical cancer). Women with CD have a 53% greater risk of developing cervical cancer when compared to normal healthy females [75, 76]. This gene, which helps mediate the formation of the stabilized collagen cross-link, also plays an important role in the progression of bone cancer [77]. It also regulates the expression of Hexokinase 2 through STAT3 signaling pathway and promotes colorectal cancer [78]. Epigenetic modifications affect the transcriptional state of the gene in which lysine acetylation, one of the most common post-translational modifications, plays an important role in many factors, such as inflammatory response, cancer, and even addiction, as well as a catalog of other medical conditions [74, 79-81]. In a 2011 study conducted by Loukia G Tsaprouni and his colleagues, the association of lysine acetylation with inflammatory gene regulation (which has implications for the study of inflammatory bowel diseases such as Crohn’s Disease and Ulcerative colitis) was explored. The team used two animal models to investigate its role in which results pertaining to Crohn’s disease obtained showed a slight induction of acetylation on H4 in the non-inflamed ileum but a significant elevation in the inflamed tissues. So, acetylation of lysine also plays a major part in the inflammatory response [80]. Thus, inhibiting intracellular posttranslational modifications directly affects collagen formation and fibrosis. However, the PLOD2 gene is essential for human physiology, so using this gene as a drug target should be specific and ideal for the fibrosis-affected area so that it does not cause any severe side effects.

ZNF165 (Zinc Finger Protein 165) is a member of the Kruppel family of zinc finger proteins. Members of this DNA-binding protein family act as transcriptional regulators, expressed in human adult testis and located within a cluster of zinc finger family members [82]. Colorectal cancer is one of the most common malignant complications observed in most inflammatory bowel disease patients, among which 80 patients with CD or ulcerative colitis (UC) have developed colorectal cancer in later stages. This is due to chronic inflammation of the colon that has increased the risk of developing colon cancer, in which the constant turnover of the intestinal lining’s cells increases the chance of abnormalities and cancer in CD and UC patients [83, 84]. Dong and his colleagues in the year 2004 have found that ZNF165 mRNA is expressed in tumors of various tissue, which was confirmed by RT–PCR, real-time PCR, and Northern blotting analysis in which its gene expression was found in hepatocellular carcinoma, gastric cancer, colon cancer, and non-small-cell lung carcinoma [84]. Studies have also reported that ZNF165 is frequently expressed in human urinary bladder transitional cell carcinoma, which is confirmed as a novel diagnostic biomarker and a vaccine target in urinary bladder cancer [85]. ZNF165 activates the T signaling pathway [86, 87]. This activates Smad2 and Smad3. This results in forming of heteromeric complexes (Smad2/3/4) that enter the nucleus to regulate transcription. These cytokines (Transforming growth factor-betas) are expressed in the colon and act as tumor suppressors and tumor promoters during colorectal cancer. Studies also show that colorectal cancers are resistant to the tumor suppressor effects of Transforming growth factor-beta [88]. Thus, colon cancer is associated with the TGFβ signaling pathway, which is activated by the ZNF165 gene. Also, its expression is predominant in most cancers, suggesting that ZNF165 may serve as a novel diagnostic biomarker and vaccine target in cancer as well as CD.

BOK (BCL-2–related ovarian killer) belongs to the BCL2 family of proteins. The proteins in this family act as pro-apoptotic or antiapoptotic regulators that play an important role in many cellular processes that function through different apoptotic signaling pathways [89-91]. BOK acts as a pro-apoptotic protein that positively regulates the intrinsic apoptotic process in a BAX- and BAK1-dependent manner or a BAX- and BAK1-independent manner [90-92]. Any alterations in these apoptotic signaling pathways result in several human diseases, including cancer, viral infections, neurodegenerative disorders, AIDS, and autoimmune diseases [93]. Chronic activation of lamina propria T lymphocytes (LPL) in the intestinal mucosa region has led to an uncontrolled and unnecessary inflammatory reaction in CD patients. Thus, the LPL apoptosis pathway was found to be disturbed by a defect in the intrinsic downregulatory mechanism, which controls the apoptosis of LPL, leading to their inappropriate accumulation resulting in chronic inflammation in CD patients [94]. NOD genes recognize intestinal luminal bacterial antigens that activate an extreme and exaggerated adaptive immune response that induces mucosal inflammation leading to bowel damage [95]. The NOD2 gene is found to be co-expressed with BCL-2A1 (antiapoptotic member of the BCL-2 family), which has a physical interaction with the BOK gene [96, 97]. However, further studies must confirm BOK gene involvement in the LPL apoptosis pathway. On the other hand, studies also report that stage II and III colorectal cancer (malignant complication observed in CD) patients show a decreased level of BOK protein in their tumors compared to the normal tissues, and increased levels are associated with earlier colorectal cancer recurrence and reduced overall survival. So, the BOK gene is studied as a prognostic marker in colorectal cancer [98]. This literature evidence confirms that the BOK gene is involved in apoptosis, cancer, and inflammation in the colon, which can serve as a diagnostic marker gene in CD patients.

Armadillo repeat-containing X-linked 4 (ARMCX4) is a protein-coding gene that belongs to the armadillo family. This family of armadillo proteins (ARMCX)1-6 are primarily implicated in embryogenesis and tumorigenesis mechanisms. Cervical cancer is the second most common cancer in women worldwide and is said to have some association with inflammatory bowel disease (IBD). Hutfless and his colleagues, in the year 2008, hypothesized that inflammatory bowel disease (IBD), an immune-related condition treated with immune-modulating medications, could increase susceptibility to cervical cancer [99]. Studies on extraintestinal cancers in IBD showed an increased risk of cervical cancer in CD and not in Ulcerative colitis (UC) patients. They propose that IBD medications could increase the risk of cervical cancer [99]. On the other hand, Chang and his colleagues, in the year 2017, performed whole exome sequencing and identified novel mutations in several potential cancer drivers and passenger cancer genes ARMCX4 gene is one among them, which is responsible for causing endometrial cancer. It is stated as non-canonical cancer-related gene [100]. Thegene–gene interaction network constructed in this study shows ARMCX4 gene is co-expressed along with ZNF165 and STAT3 genes, which plays a vital role in developing colon cancer and inflammation in CD patients in which activation and mutation of the ARMCX4 gene along with other cancer driver and passenger cancer genes results in endometrial cancer. Though previous studies report very little information about this gene but its role in tumorigenesis is confirmed, which in turn is associated with IBD, thus ensuring it may serve as a diagnostic marker in evaluating the severity of tumorigenesis concerning CD patients by precisely targeting the pathological mechanisms it could also serve as an exciting drug target.

CX3CR1 (C-X3-C Motif Chemokine Receptor 1) is a receptor for the C-X3-C chemokine fractalkine (CX3CL1). It is also known as G protein-coupled receptor 13 (GPR13) and fractalkine receptor. This receptor is found on early leukocyte cells whose activation results in many diverse functions in human physiology. When ligand (CX3CL1) binds to the receptor (C-X3-C Motif Chemokine Receptor 1), it activates many signaling mechanisms such as immune response, inflammation, cell adhesion, and chemotaxis [101-104]. CX3CR1+ macrophages protrude outside the epithelial cell layer to sense the bacteria in which the endocytosed bacteria are digested and released, which are taken up by CD103^+^ dendritic cells and presented to T- cell *via* antigen-MHC complex. Activated T-cells in the lamina propria will kill the bacteria to protect the host from any antigen or foreign particle invasion. Thus, CX3CR1+ macrophages are very important for pathogenic microbe elimination, and also experiments on mice showed that CX3CR1 deficient mice suffer from severe colitis [105]. Studies also suggest that missense mutation in the gene encoding CX3CR1 leads to impaired antifungal responses in CD patients [106]. Thus, CX3CR1+ macrophage is crucial for maintaining gut integrity [107]. It also plays a vital role in controlling abnormal inflammation in the intestine, thereby preventing tissue damage [105]. When ligand CX3CL1/fractalkine binds to the CX3CR1 receptor, it induces upregulation in heme-oxygenase 1 (HMOX1) signaling pathway in macrophages *via* STAT3 phosphorylation which results in anti-inflammation and immunomodulatory effects which confirms that STAT3/HMOX1 axis serves as an important pathway that mediates the regulatory function of CX3CR1+ macrophages [108]. Increased CX3CL1/CX3CR1 in human and mouse models suggested that it could be a potential biomarker for colitis [107], and V249I polymorphism of the CX3CR1 gene is associated with intestinal strictures, particularly in smokers in CD patients [109]. Studies report that CX3CR1 is also involved in regulating the tumor inflammatory microenvironment and serves as a protective biomarker in colorectal cancer [110]. Thus, targeting CX3CR1 could be a powerful and efficient approach in fighting CD. Still, at the same time, it is essential to cautiously target precise etiological and pathological mechanisms concerning the appropriate usage of the CX3CR1 gene as a drug or vaccine target.

## 5 CAVEATS

The surgical margins are often used as “control” tissues and may appear “normal” by histopathological analysis, however, the changes at molecular and cellular levels might have occurred which have not been characterized.

## CONCLUSION

To conclude, Recursive feature elimination (RFE), a machine learning approach along with DEG analysis, has resulted in identifying five biomarker genes, PLOD2, ZNF165, BOK, CX3CR1, and ARMCX4, and the role of these biomarkers helps in the early prediction of postoperative recurrence in CD patients. Also, this study strengthens previous literature sources confirming that these biomarkers are linked with CD in pathway-level-inducing inflammation in the colon and intestine. On the other hand, these biomarkers are also reported to be connected with tumor confirming their role in causing cancer, inflammation, and immunity. WGCNA analysis demonstrates that tobacco use doubles the risk of disease recurrence in both sexes. Thus, present advances focus on developing improved strategies to prevent disease recurrence. These biomarkers may indicate a new direction for early diagnosing and treating postoperative recurrence in CD.

## Data Availability

Dataset GSE186582 can be downloaded from the Gene Expression Omnibus database (GEO, https://www.ncbi.nlm.nih.gov/geo/).

## LIST OF ABBREVIATIONS

BH: BCL-2 Homology
CD: Crohn's Disease
DT: Decision Tree Classifier
KNN: K-nearest Neighbor
LR: Logistic Regression
RF: Random Forest Classifier
RFE: Recursive Feature Elimination
SVM: Support Vector Machine
WGCNA: Weighted Gene Co-expression Network Analysis
