# Supplementary material for: Identification of Potential Genes and Critical Pathways in Postoperative Recurrence of Crohn’s Disease by Machine Learning And WGCNA Network Analysis
Source: Curr Genomics. 2023 Oct 27;24(2):84–99. doi: 10.2174/1389202924666230601122334 (PMC10662376; doi:10.2174/1389202924666230601122334)
Supplement: Supplementary file 1 [file CG-24-84_SD1.zip › CG-24-84_SD1/3b-BMS-CG-2023-13 Supplementary Material (Figures).pdf]

Supplementary Material

Identification of Potential Genes and Critical Pathways in Postoperative Recurrence of Crohn's Disease by Machine Learning And WGCNA Network Analysis

Aruna Rajalingam[1], Kanagaraj Sekar[2] and Anjali Ganjiwale[1,*]

[1]Department of Life Sciences, Bangalore University, Bangalore, Karnataka, 560056, India; [2]Laboratory for Structural Biology and Bio-computing, Computational and Data Sciences, Indian Institute of Science, Bangalore, Karnataka, 560012, India

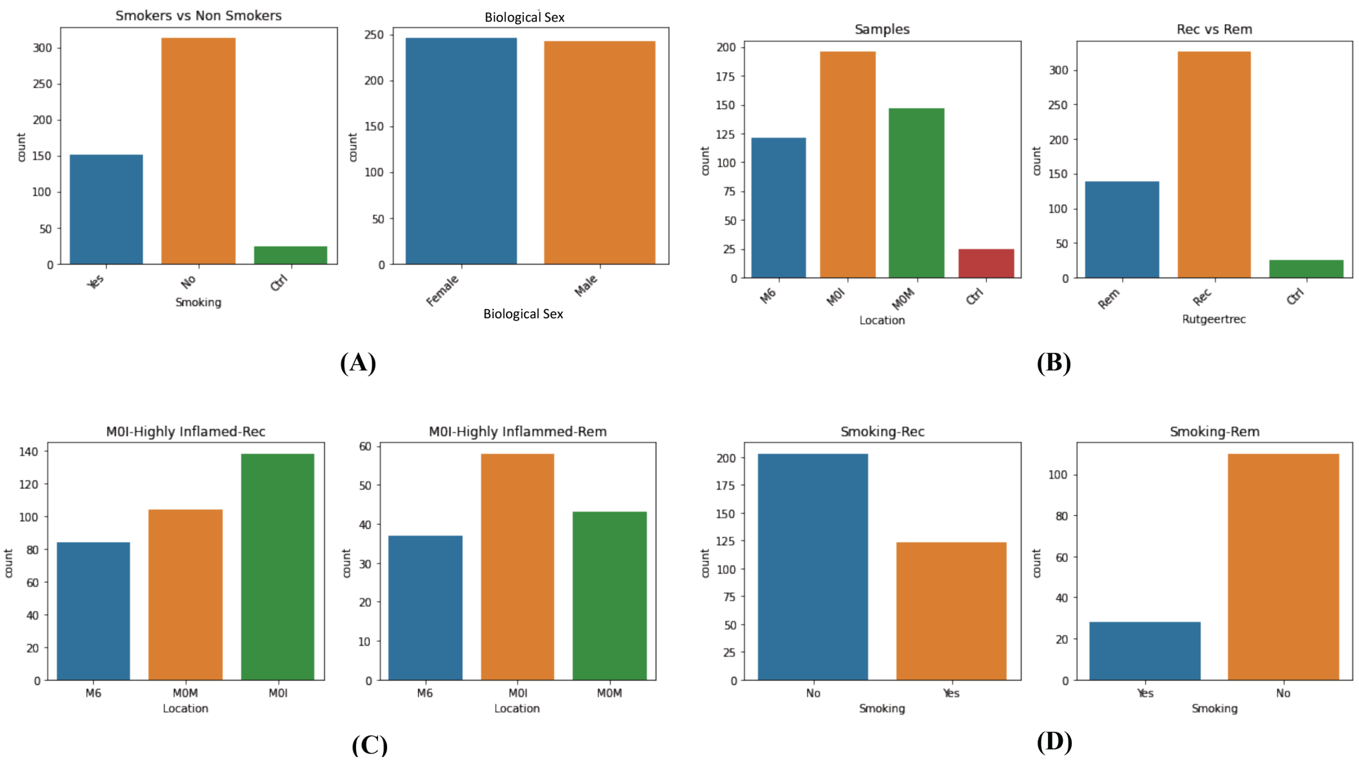

Fig. (S1). Preliminary data analysis on GSE186582 dataset showed (A)Equal distribution of males and females containing both smokers and non-smokers (B) More inflamed ileum (M0I) compared to other samples (M0M, M6, Ctrl) with increased amount of recurrence samples when compared to remission samples (C) Recurrence and remission samples showed more inflamed ileum (M0I) samples (D) Recurrence samples have more smokers compared to remission samples.

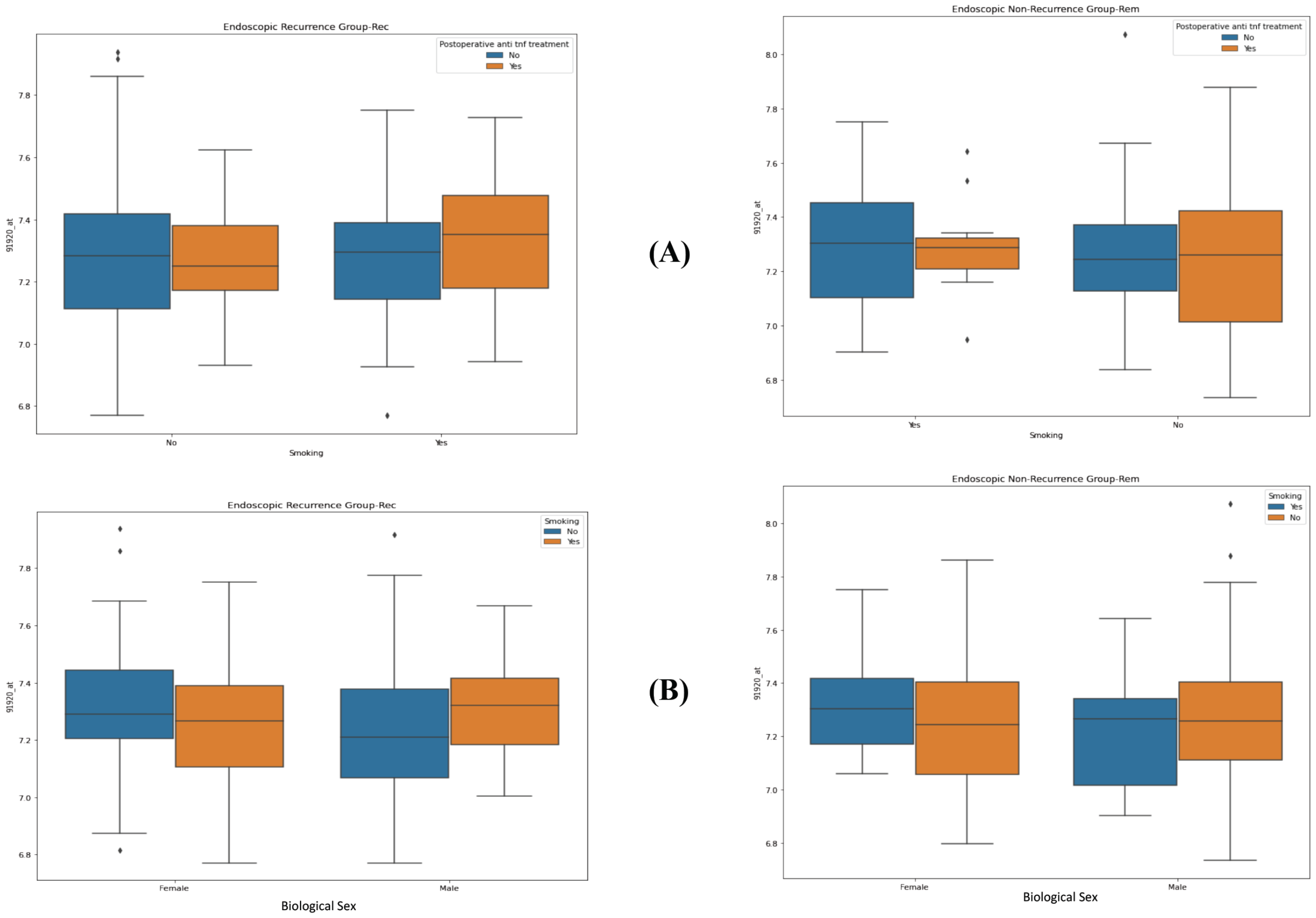

Fig. (S2). Preliminary data analysis on GSE186582 dataset showed (A) Post-operative anti-TNF treatment infers that smoking is associated with the risk of recurrence and reduces the chance of remission in non-recurring groups (B) Smoking increases the risk of recurrence in males. In contrast, smoking does not induce recurrence in females compared to males. In remission or non-recurring groups, smoking does not bring any changes in both males or females.

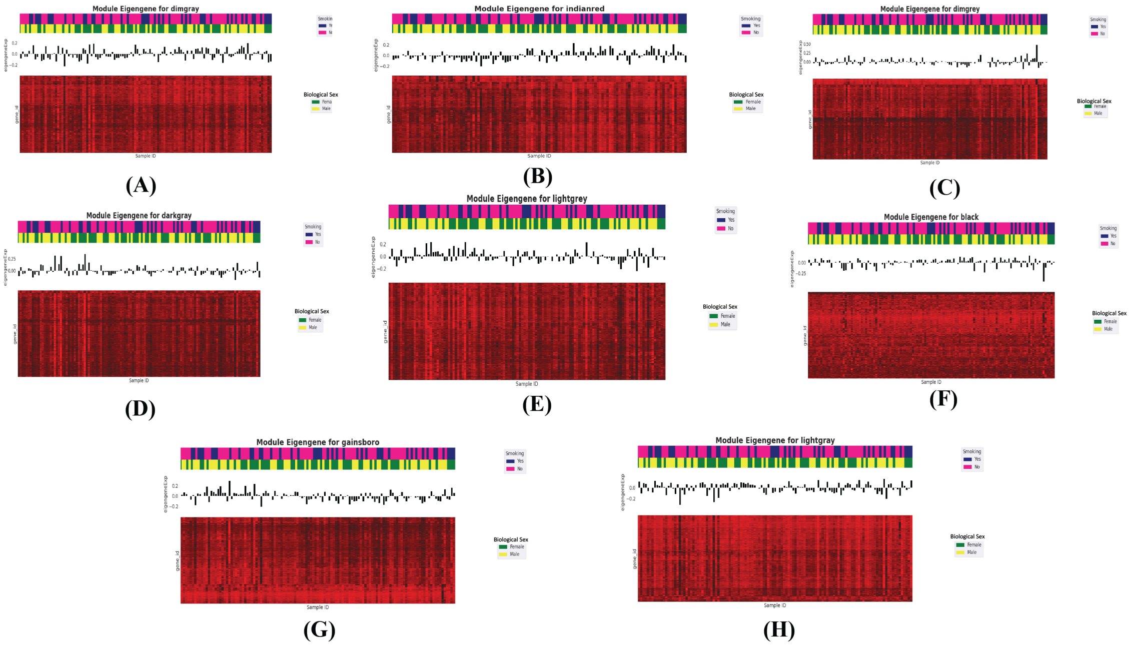

Fig. (S3). Heatmap of Module Eigengene showing the overall expression of eigengenes present in the module in (A) Dimgray (B) Indianred (C) Dimgrey (D) Darkgray (E) Lightgrey (F) Black (G) Gainsboro (H) Lightgray.

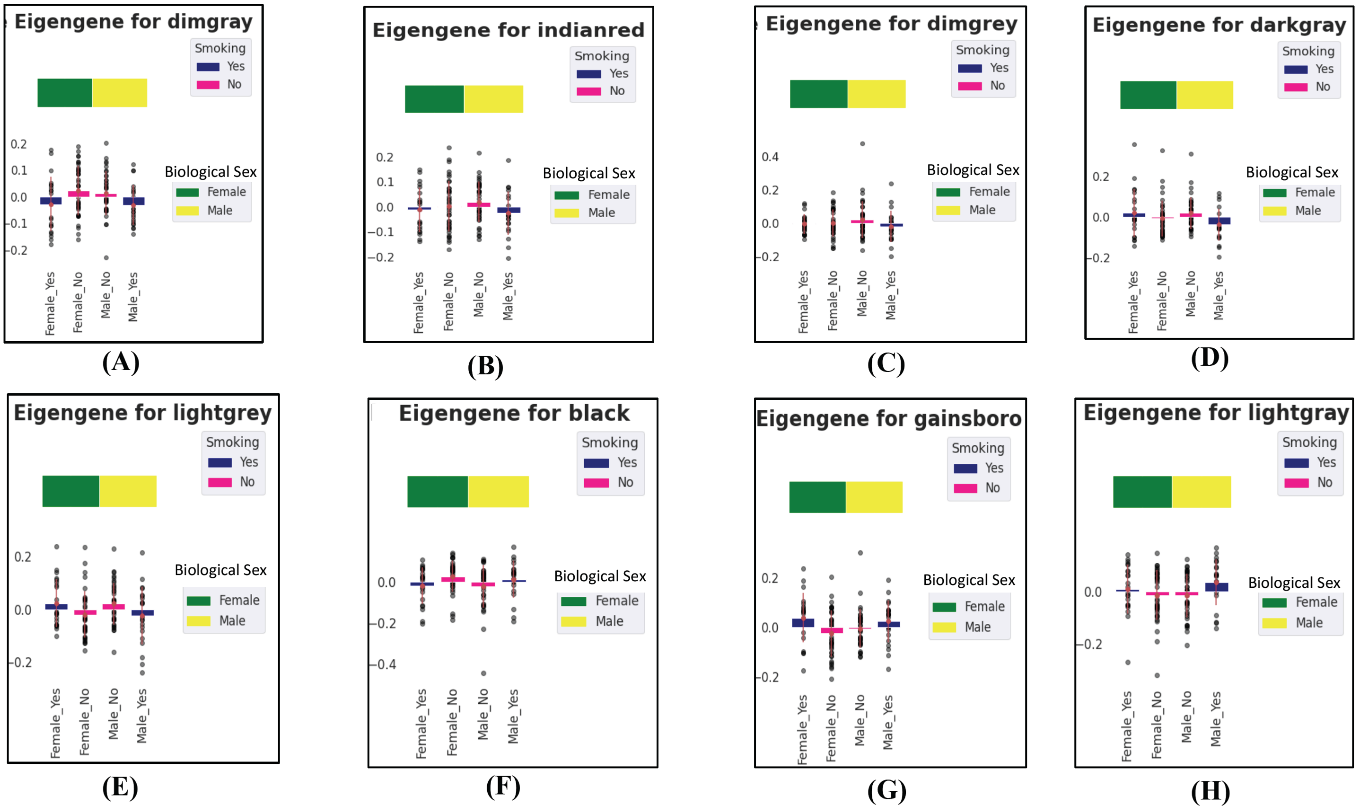

Fig. (S4). Plots showing the correlation of each module association with smoking concerning both sex distribution for **(A)** Dimgray **(B)** Indianred **(C)** Dimgrey **(D)** Darkgray **(E)** Lightgrey**(F)** Black**(G)** Gainsboro and **(H)** Lightgray.

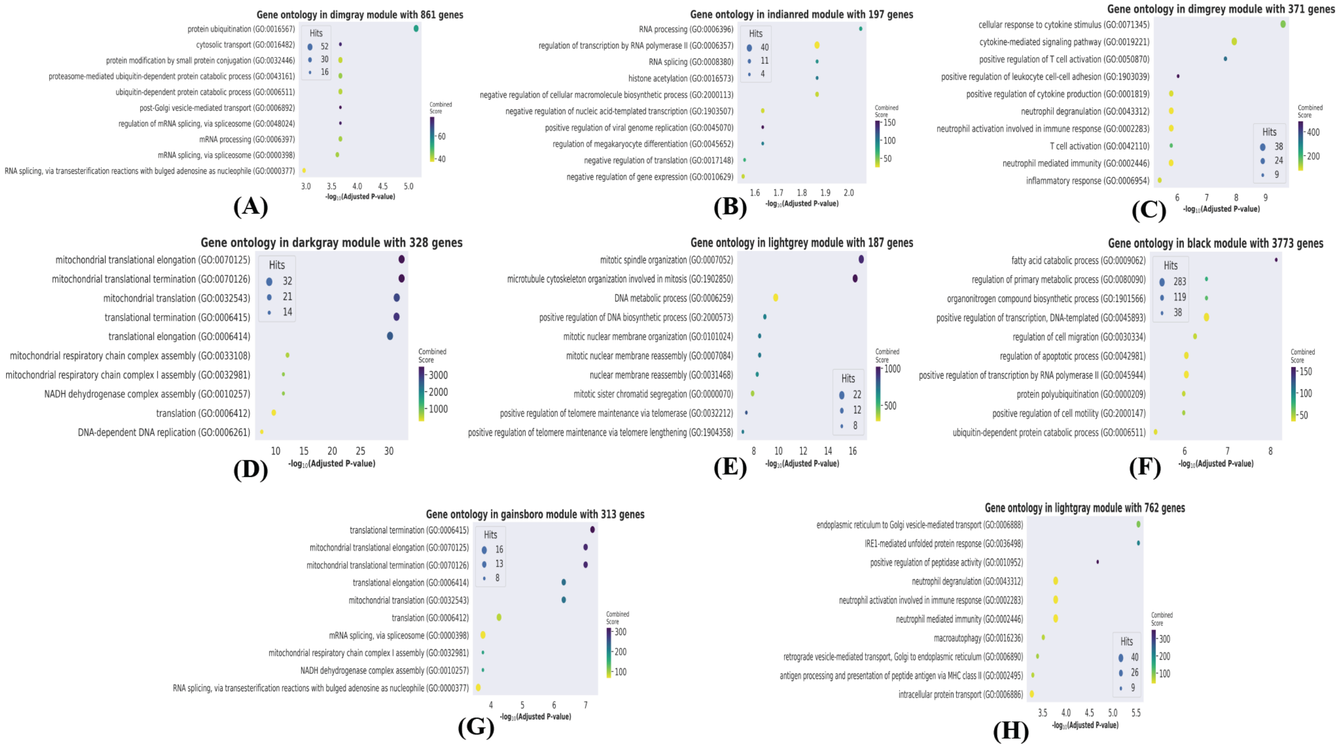

Fig. (S5). Gene ontology (GO) enrichment analysis for **(A)** Dimgray **(B)** Indianred **(C)** Dimgrey **(D)** Darkgray **(E)** Lightgrey **(F)** Black **(G)** Gainsboro, and **(H)** Lightgray.

CLASSIFICATION WITHOUT FEATURE SELECTION

	Model	Fitting time	Scoring time	Accuracy	Precision	Recall	F1_score	AUC_ROC
2	Support Vector Machine	0.211027	0.144176	0.666667	0.333333	0.500000	0.533922	0.640675
1	Decision Tree	0.300237	0.141497	0.645556	0.589762	0.610714	0.641003	0.610714
4	K-Nearest Neighbors	0.115020	0.197901	0.634444	0.406667	0.491071	0.541552	0.568056
0	Logistic Regression	0.361426	0.239932	0.626667	0.513810	0.535119	0.596241	0.611905
3	Random Forest	0.363338	0.154252	0.625556	0.431528	0.491071	0.549218	0.601389

(A)

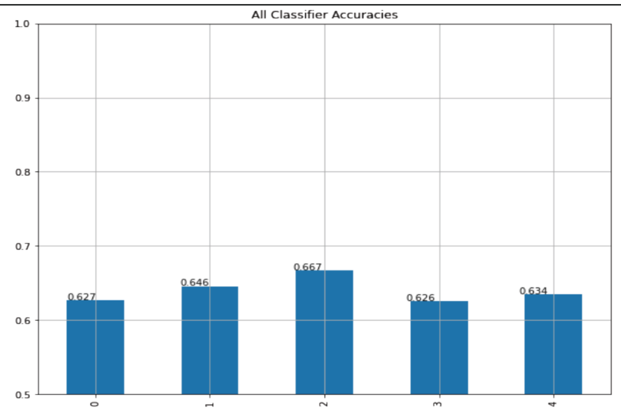

(B)

Fig. (S6). Classification accuracy without feature selection. **(A)** Each model (Logistic Regression, Random Forest, K-Nearest Neighbour, Decision Tree, Support Vector Machine) accuracy, Precision, Recall , F1_score and AUC_ROC classification for a complete dataset (Rem vs Rec) **(B)** Barplot showing selected models (classifier) accuracy.

	Model	Fitting time	Scoring time	Accuracy	Precision	Recall	F1_score	AUC_ROC
0	Logistic Regression	0.004392	0.007756	0.666842	0.333421	0.500000	0.533770	0.397436
2	Support Vector Machine	0.005165	0.006932	0.655789	0.453509	0.511447	0.566919	0.671795
4	K-Nearest Neighbors	0.002700	0.008564	0.594211	0.519863	0.513736	0.573742	0.606410
1	Decision Tree	0.012724	0.040664	0.542105	0.461730	0.493223	0.521416	0.493223
3	Random Forest	0.133309	0.027051	0.542105	0.461730	0.493223	0.521416	0.657418

(A)

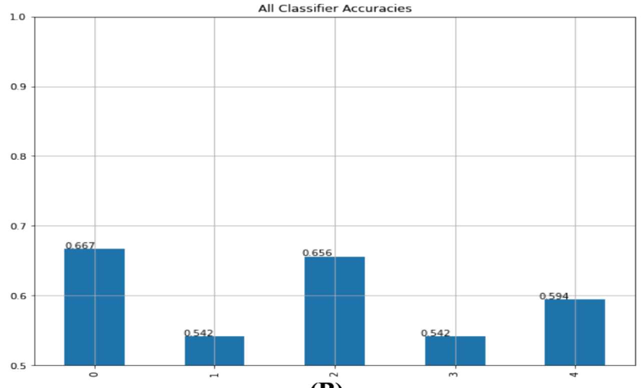

(B)

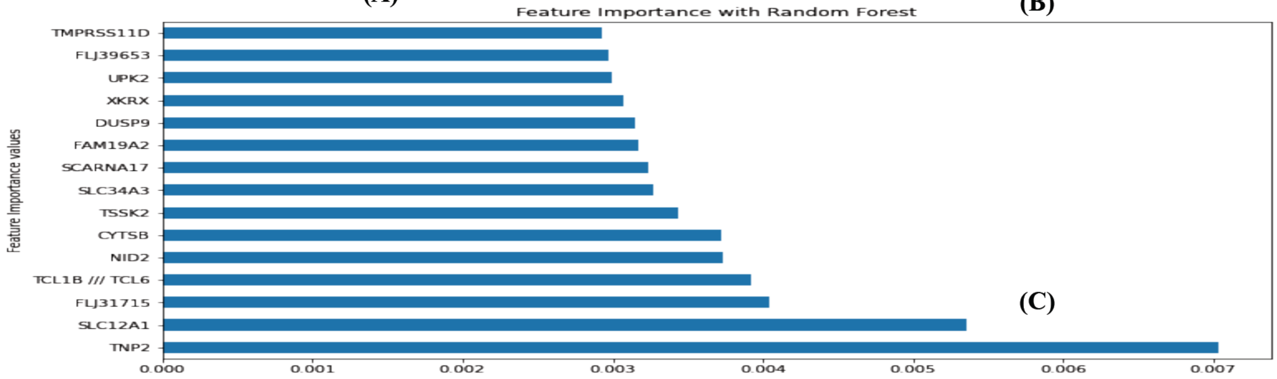

(C)

Fig. (S7). Random Forest Classifier **(A)** Each model (Logistic Regression, Random Forest, K-Nearest Neighbour, Decision Tree, Support Vector Machine) accuracy, Precision, Recall , F1_score and AUC_ROC classification based on Random Forest classifier **(B)** Barplot showing selected models (classifier) accuracy **(C)** 15 feature genes selected by Random Forest Classifier.

	Model	Fitting time	Scoring time	Accuracy	Precision	Recall	F1_score	AUC_ROC
0	Logistic Regression	0.006006	0.005613	0.812222	0.802381	0.758333	0.793183	0.911706
1	Decision Tree	0.002295	0.006606	0.790000	0.787679	0.764286	0.787483	0.764286
3	Random Forest	0.230393	0.048649	0.782222	0.807937	0.706548	0.761471	0.891964
2	Support Vector Machine	0.003940	0.006150	0.771111	0.791766	0.683333	0.740586	0.902778
4	K-Nearest Neighbors	0.002367	0.007816	0.747778	0.725357	0.717262	0.743712	0.824802

(A)

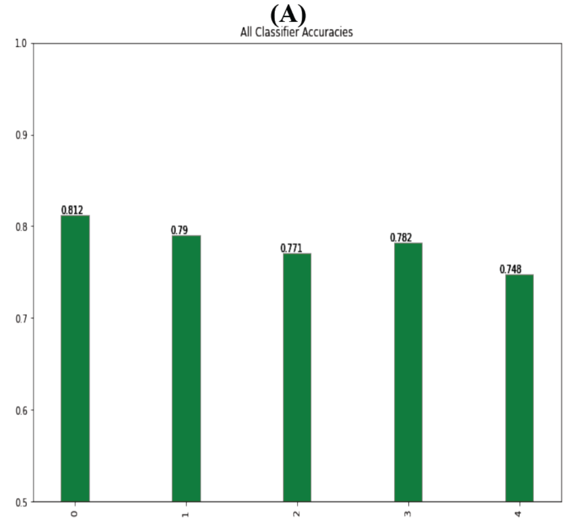

(B)

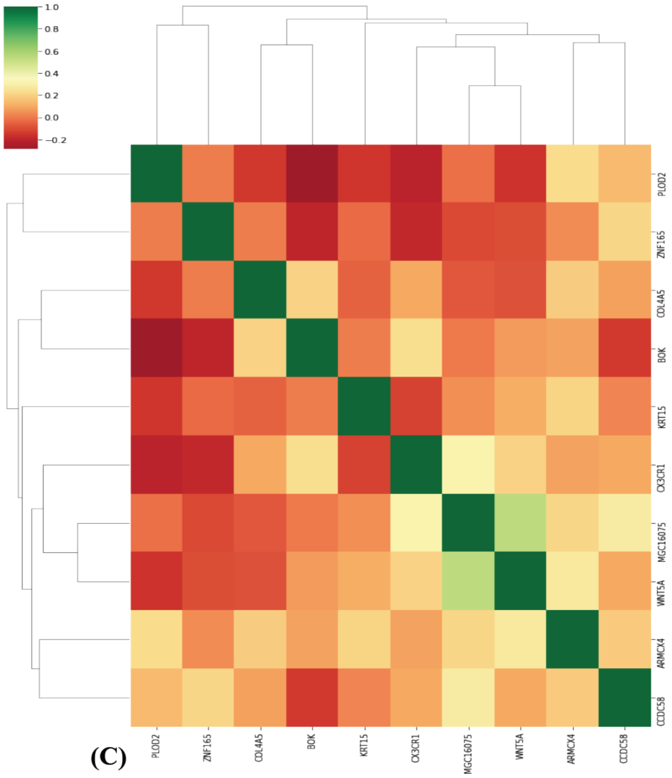

(C)

Fig. (S8). Recursive Feature Classifier **(A)** Each model (Logistic Regression, Random Forest, K-Nearest Neighbour, Decision Tree, Support Vector Machine) accuracy, Precision, Recall, F1_score, and AUC_ROC classification based on Recursive Feature Classifier **(B)** Plot showing classification accuracy of all classifiers **(C)** Cluster heatmap of 10 feature genes selected by Recursive Feature Classifier.

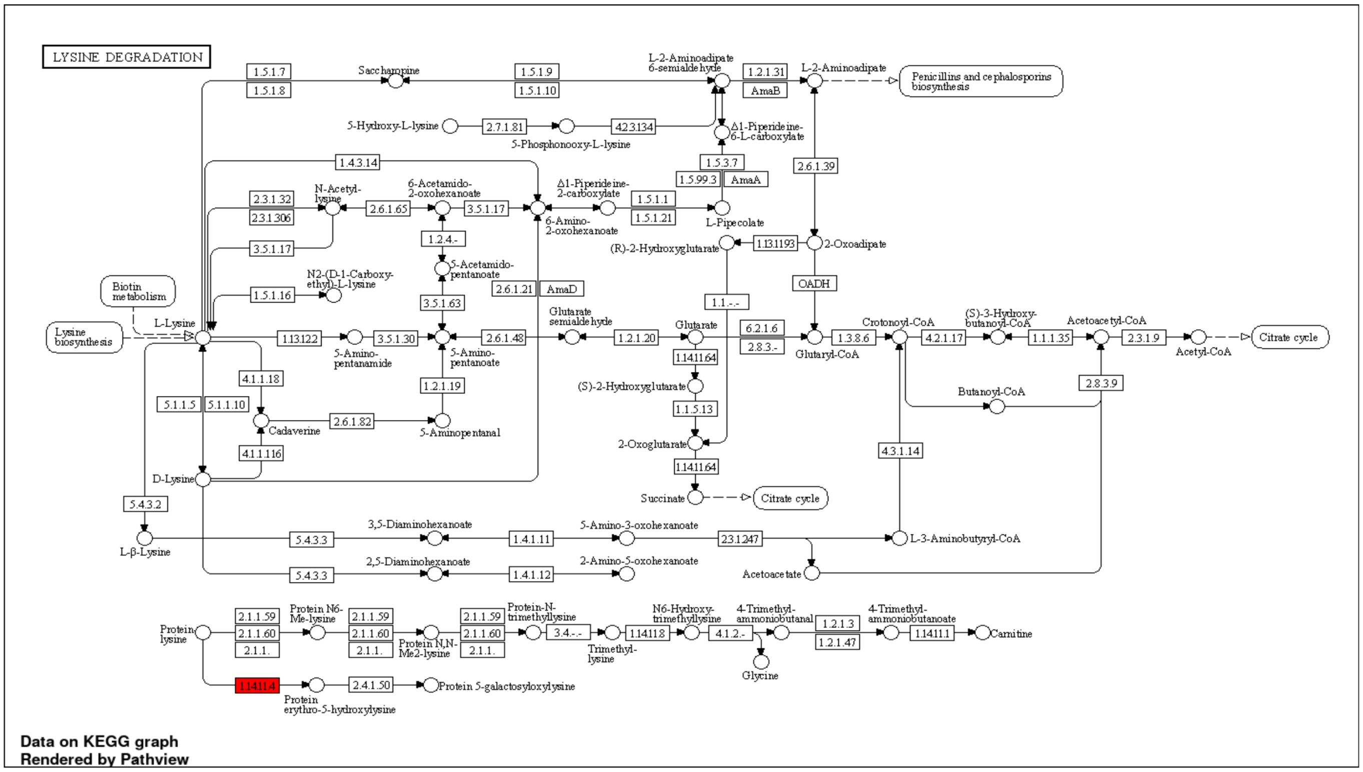

Fig. (S9). Lysine degradation pathway. PLOD2 gene (highlighted box) is involved in lysine degradation pathway. Collagen cross-link and deposition depend on lysyl hydroxylation, which is catalyzed by procollagen-lysine, 2-oxoglutarate 5-dioxygenase (PLOD). PLOD2 is the main gene critical for the stability of intermolecular crosslinks. Aberrant lysyl hydroxylation and collagen cross-link contributes to the progression of many collagen-related diseases, such as fibrosis (the main complications of Crohn's disease) and cancer.

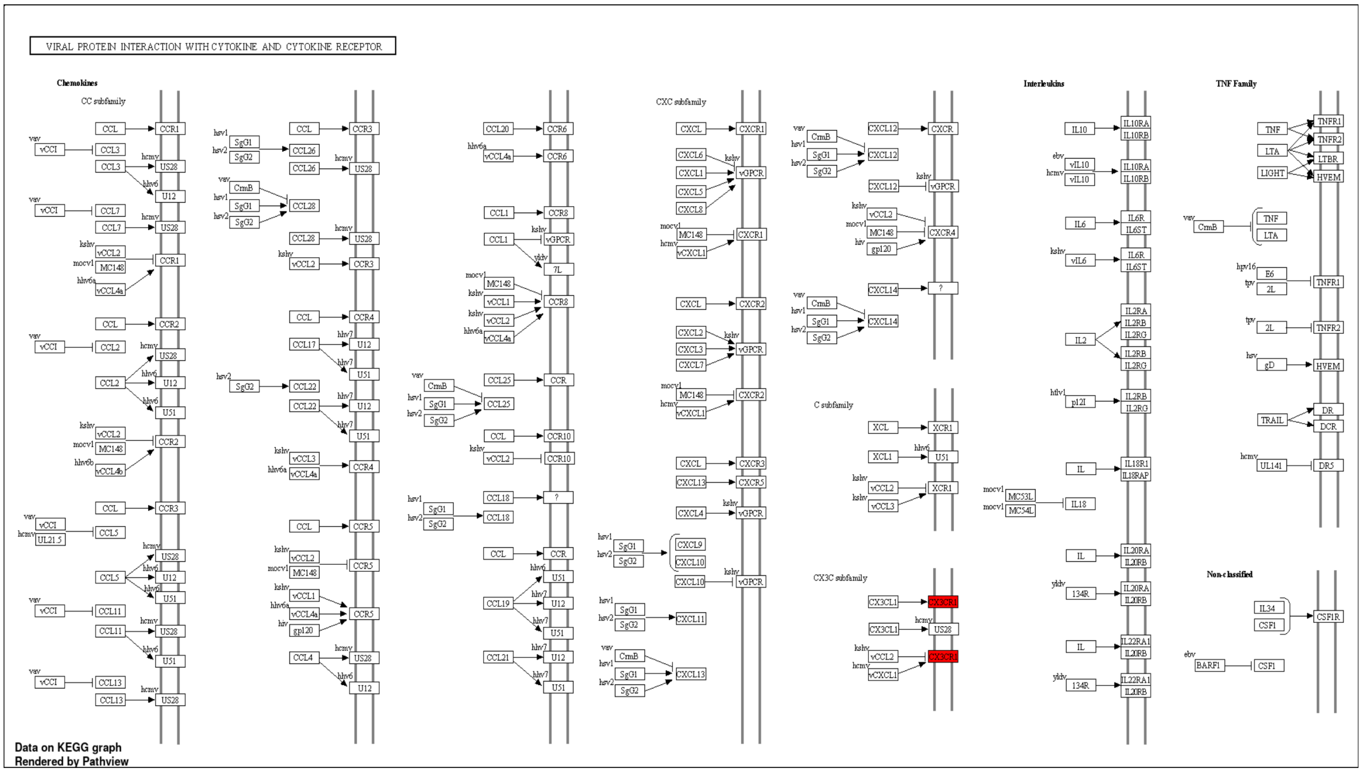

Fig. (S10). Viral protein interaction with cytokine and cytokine receptor. CX3CR1 gene (highlighted box) is involved in viral protein interaction with cytokine and cytokine receptor. CX3C motif chemokine receptor 1 (CX3CR1), also known as the fractalkine receptor or G-protein coupled receptor 13 (GPR13) which binds to the inflammatory chemokine CX3CL1 mediates migration, adhesion, and retention of leukocytes. Activated proinflammatory cytokines upregulate CX3CR1 that influence Crohn's disease (CD) phenotype and localization and enhance CD lesions.
