# Supplementary figures and images for: Identification of Potential Genes and Critical Pathways in Postoperative Recurrence of Crohn’s Disease by Machine Learning And WGCNA Network Analysis
Source: Curr Genomics. 2023 Oct 27;24(2):84–99. doi: 10.2174/1389202924666230601122334 (PMC10662376; doi:10.2174/1389202924666230601122334)

## Slide 1
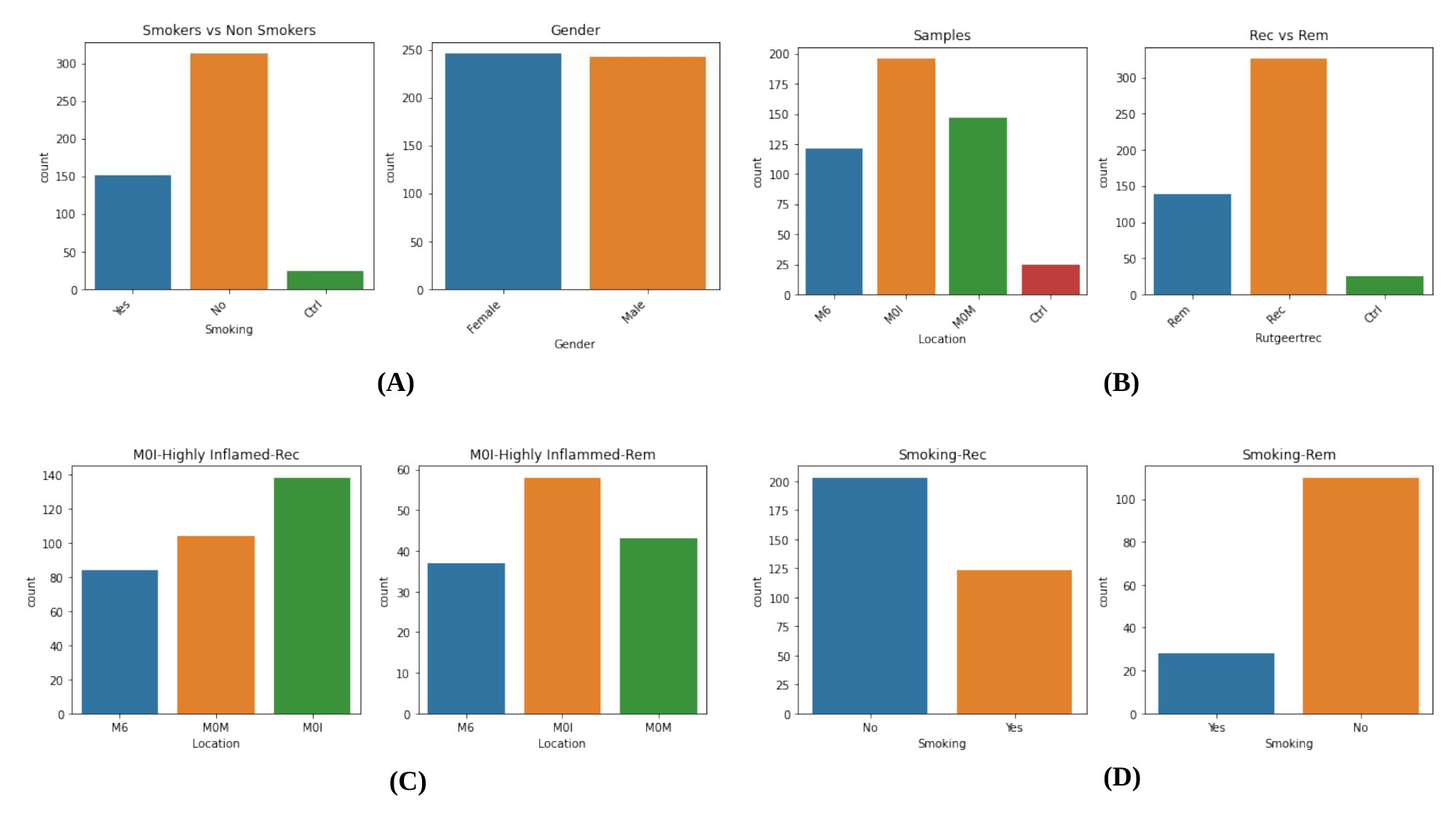

(A)
(B)
(D)
(C)

Supplement: Supplementary file 1 [file CG-24-84_SD1.zip › CG-24-84_SD1/Figure S1.pptx]

## Slide 1
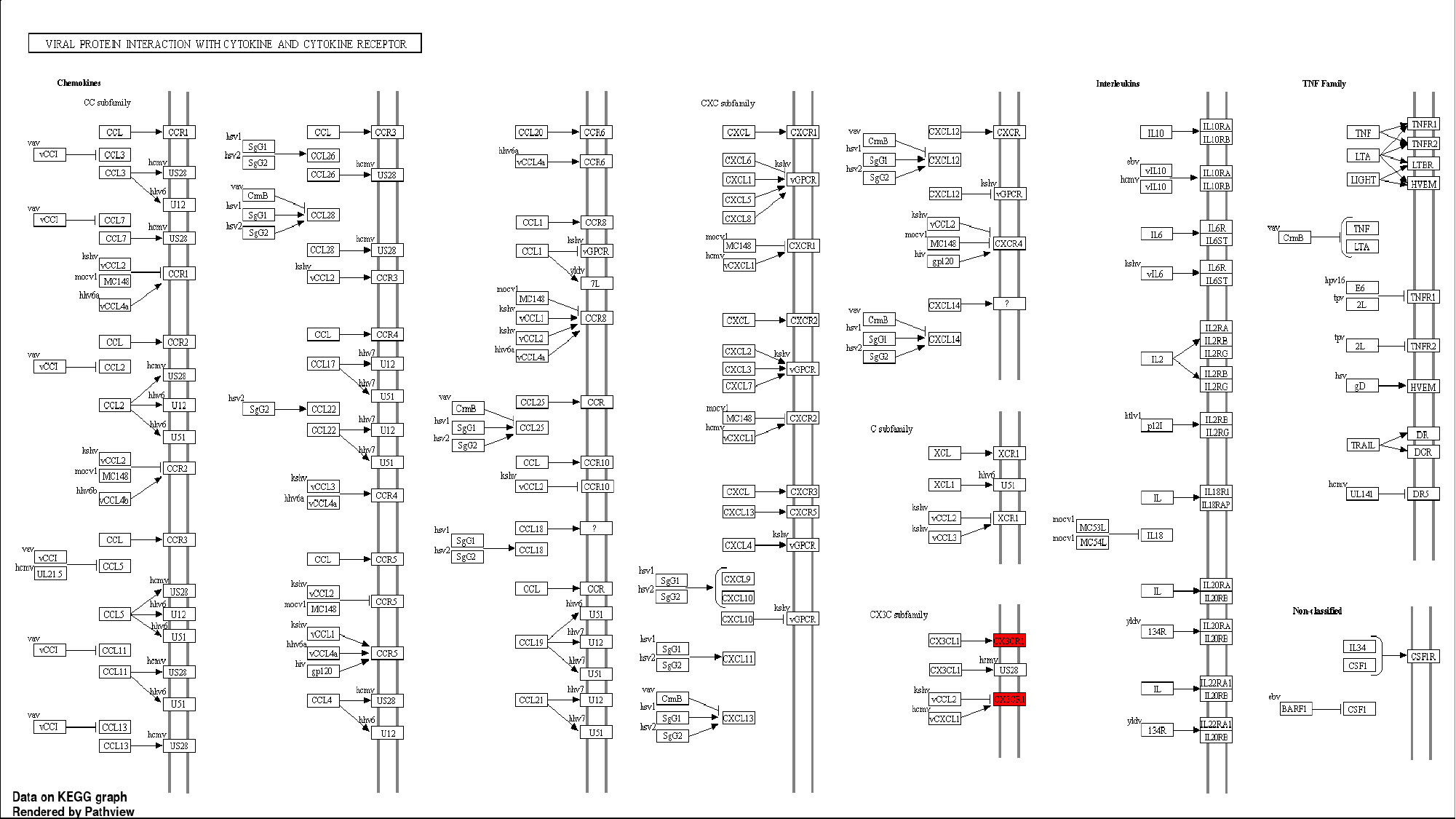

Supplement: Supplementary file 1 [file CG-24-84_SD1.zip › CG-24-84_SD1/Figure S10.pptx]

## Slide 1
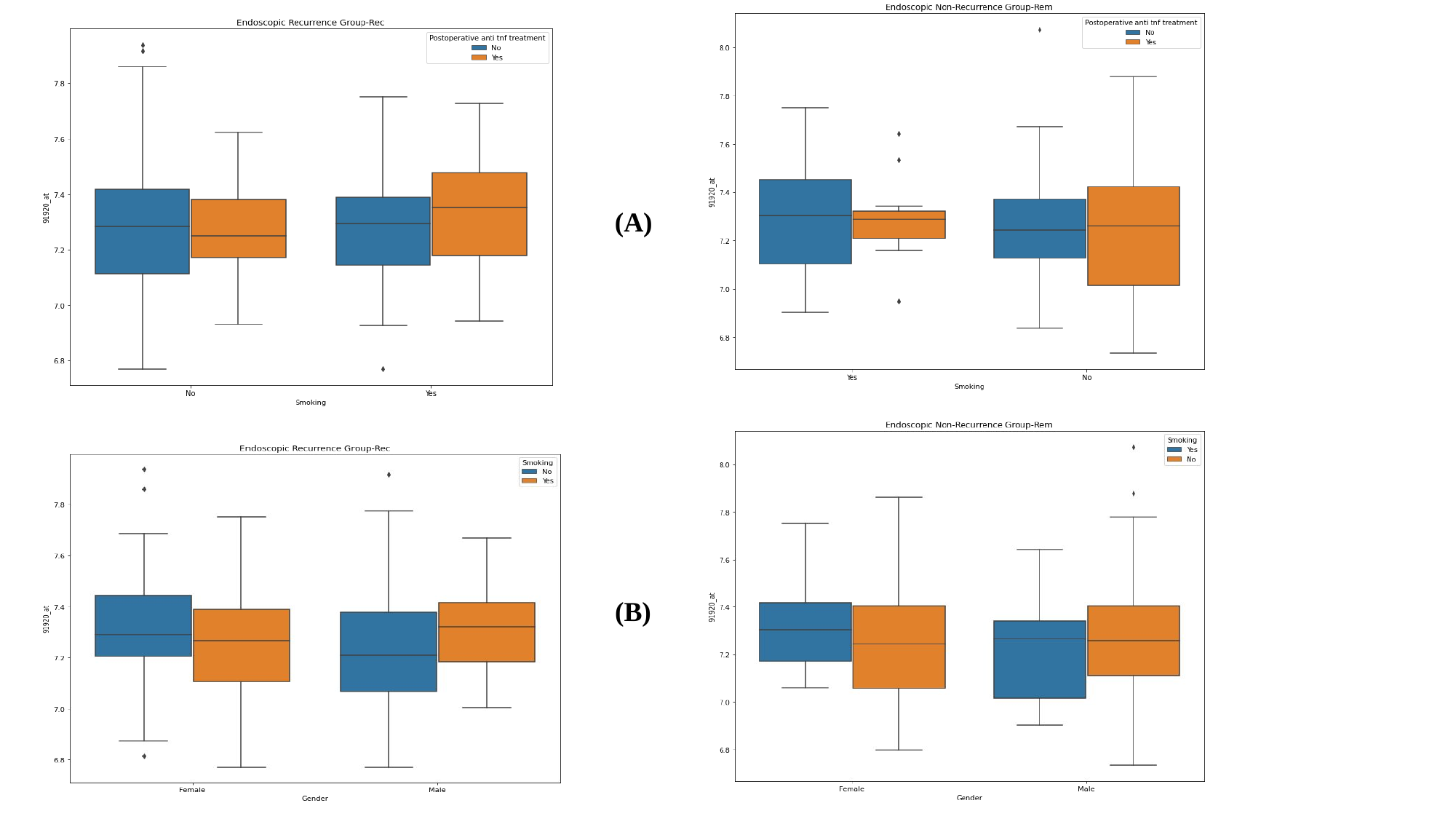

(A)
(B)

Supplement: Supplementary file 1 [file CG-24-84_SD1.zip › CG-24-84_SD1/Figure S2.pptx]

## Slide 1
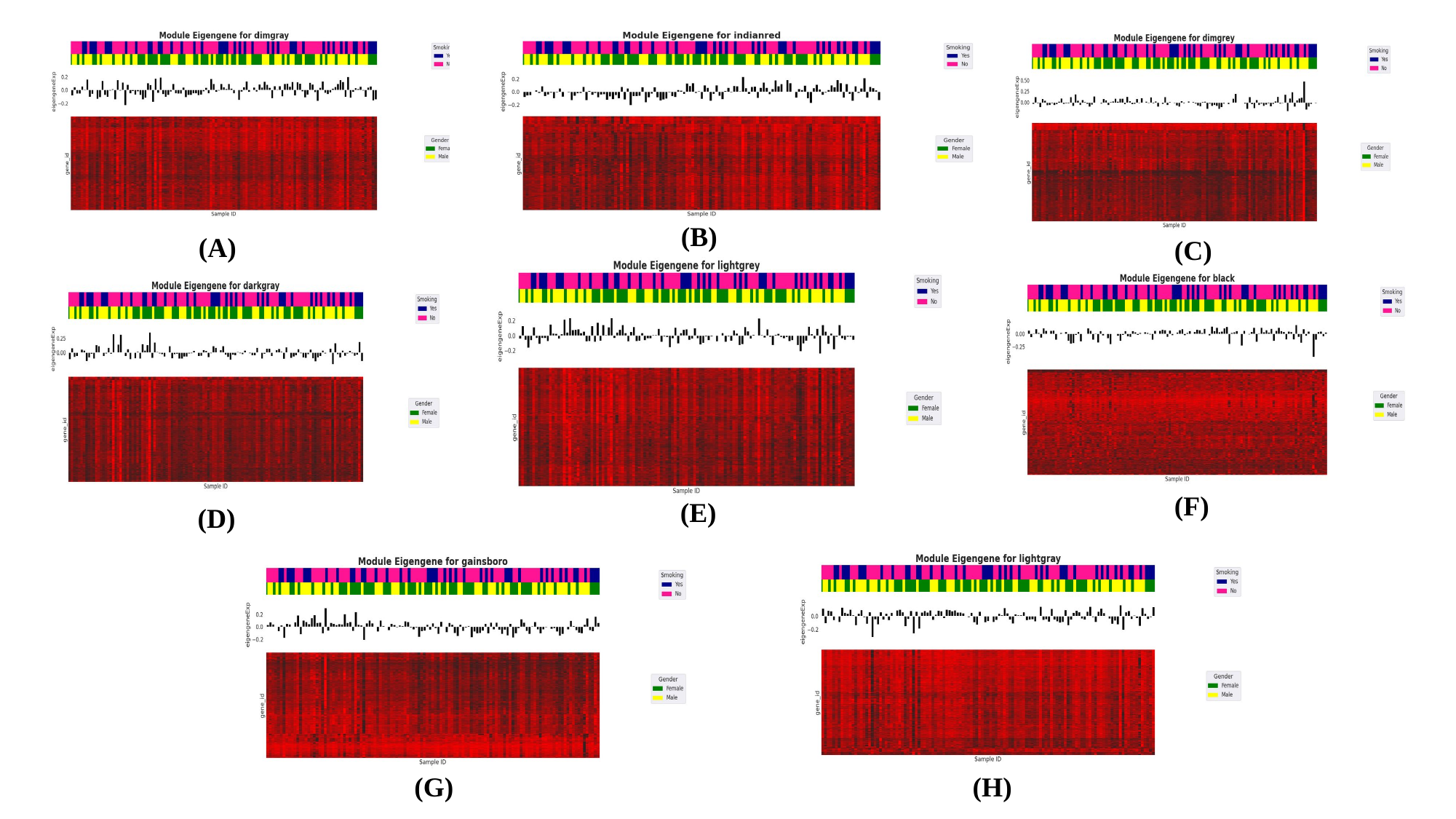

(B)
(A)
(C)
(F)
(E)
(D)
(G)
(H)

Supplement: Supplementary file 1 [file CG-24-84_SD1.zip › CG-24-84_SD1/Figure S3.pptx]

## Slide 1
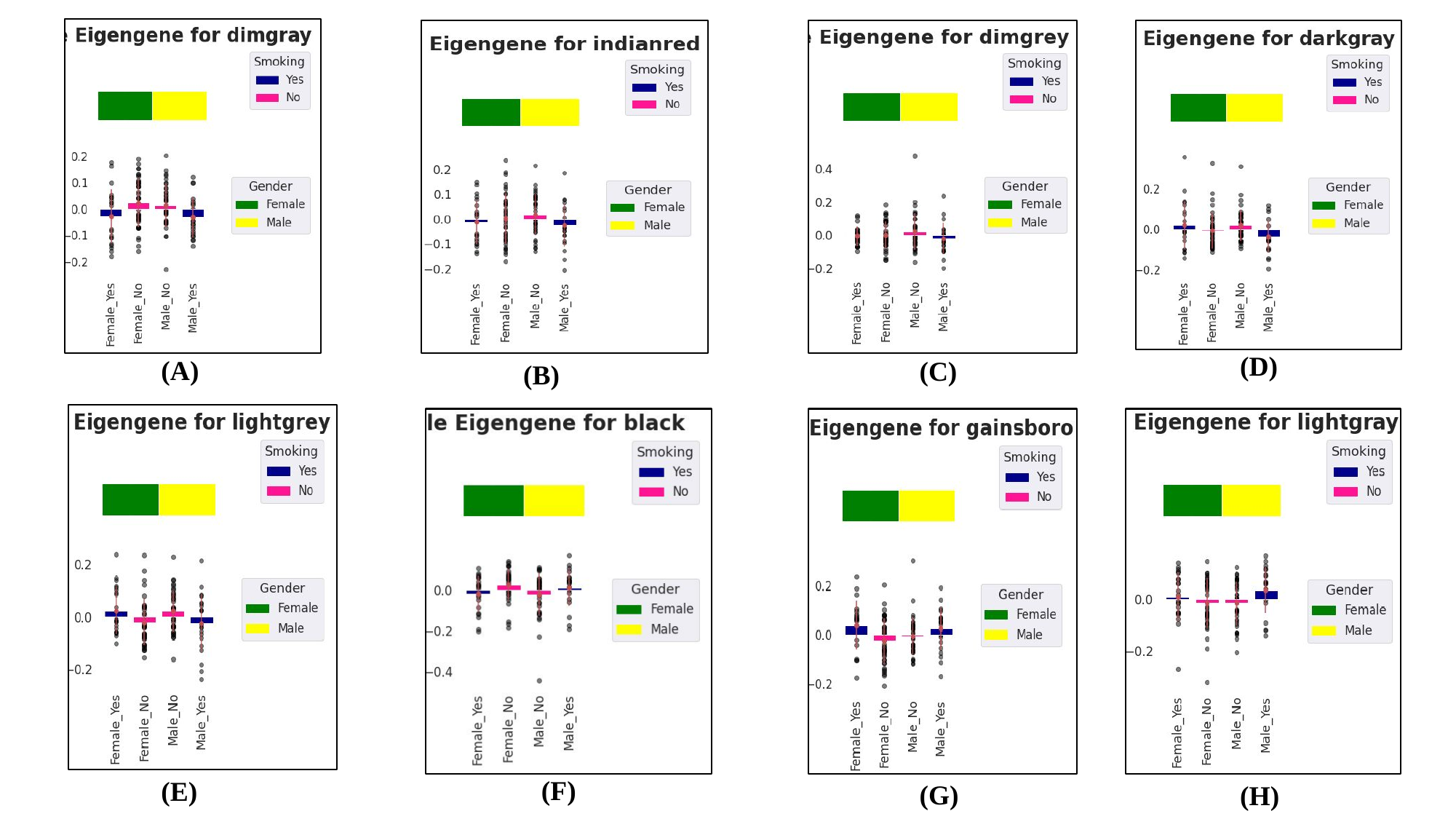

(D)
(A)
(C)
(B)
(F)
(E)
(G)
(H)

Supplement: Supplementary file 1 [file CG-24-84_SD1.zip › CG-24-84_SD1/Figure S4.pptx]

## Slide 1
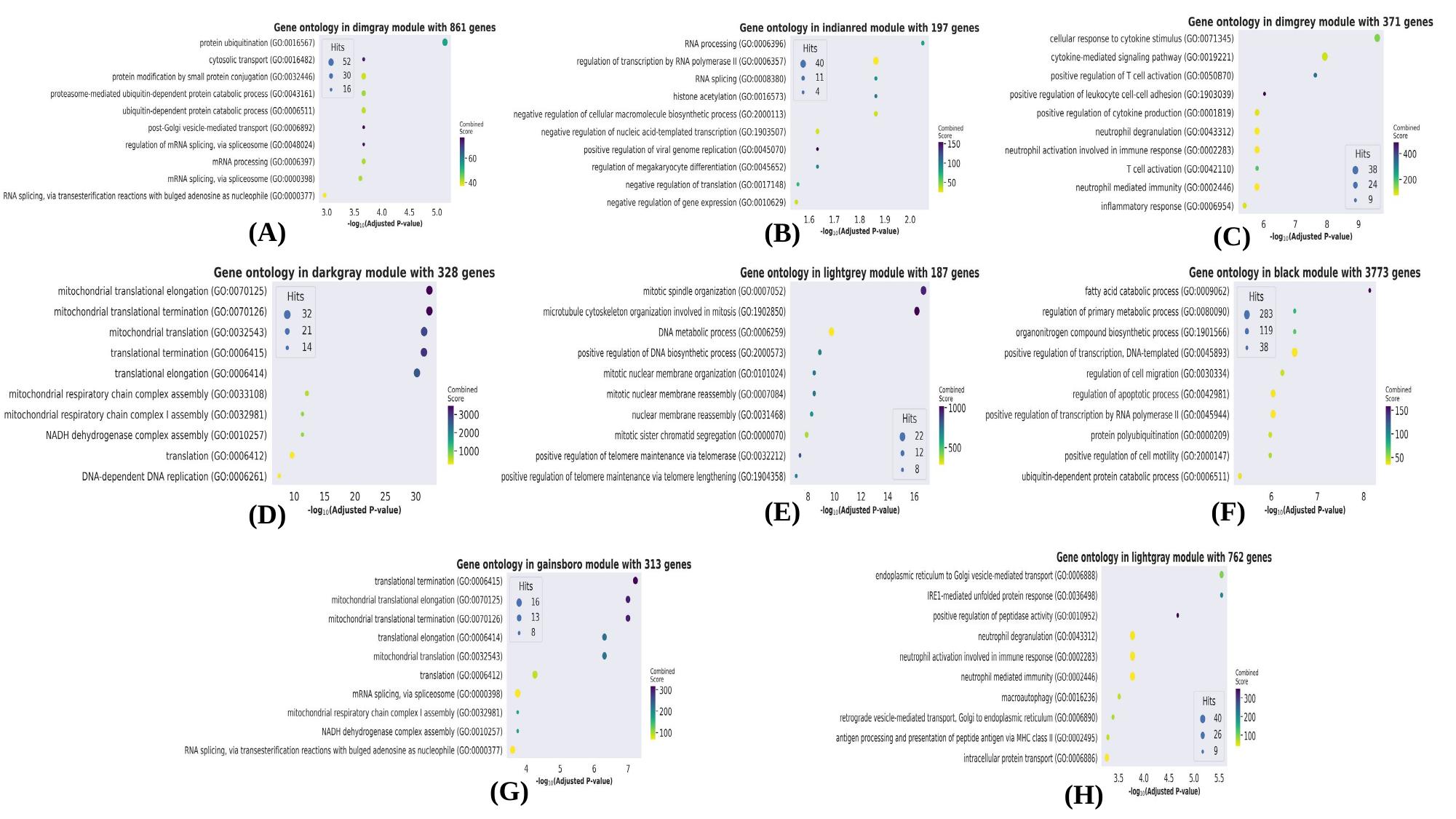

(A)
(B)
(C)
(E)
(F)
(D)
(G)
(H)

Supplement: Supplementary file 1 [file CG-24-84_SD1.zip › CG-24-84_SD1/Figure S5.pptx]

## Slide 1
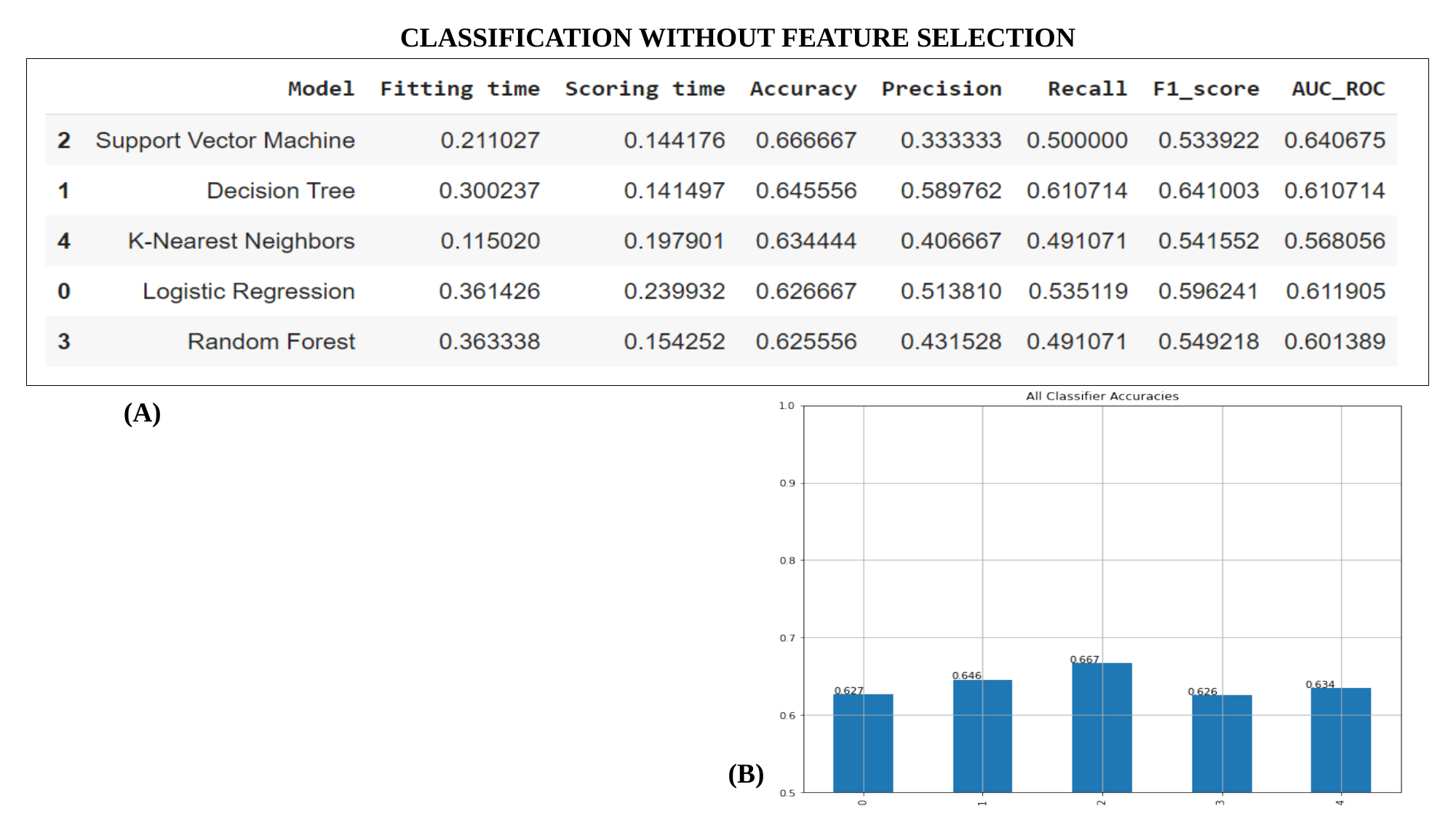

CLASSIFICATION WITHOUT FEATURE SELECTION
(A)
(B)

Supplement: Supplementary file 1 [file CG-24-84_SD1.zip › CG-24-84_SD1/Figure S6.pptx]

## Slide 1
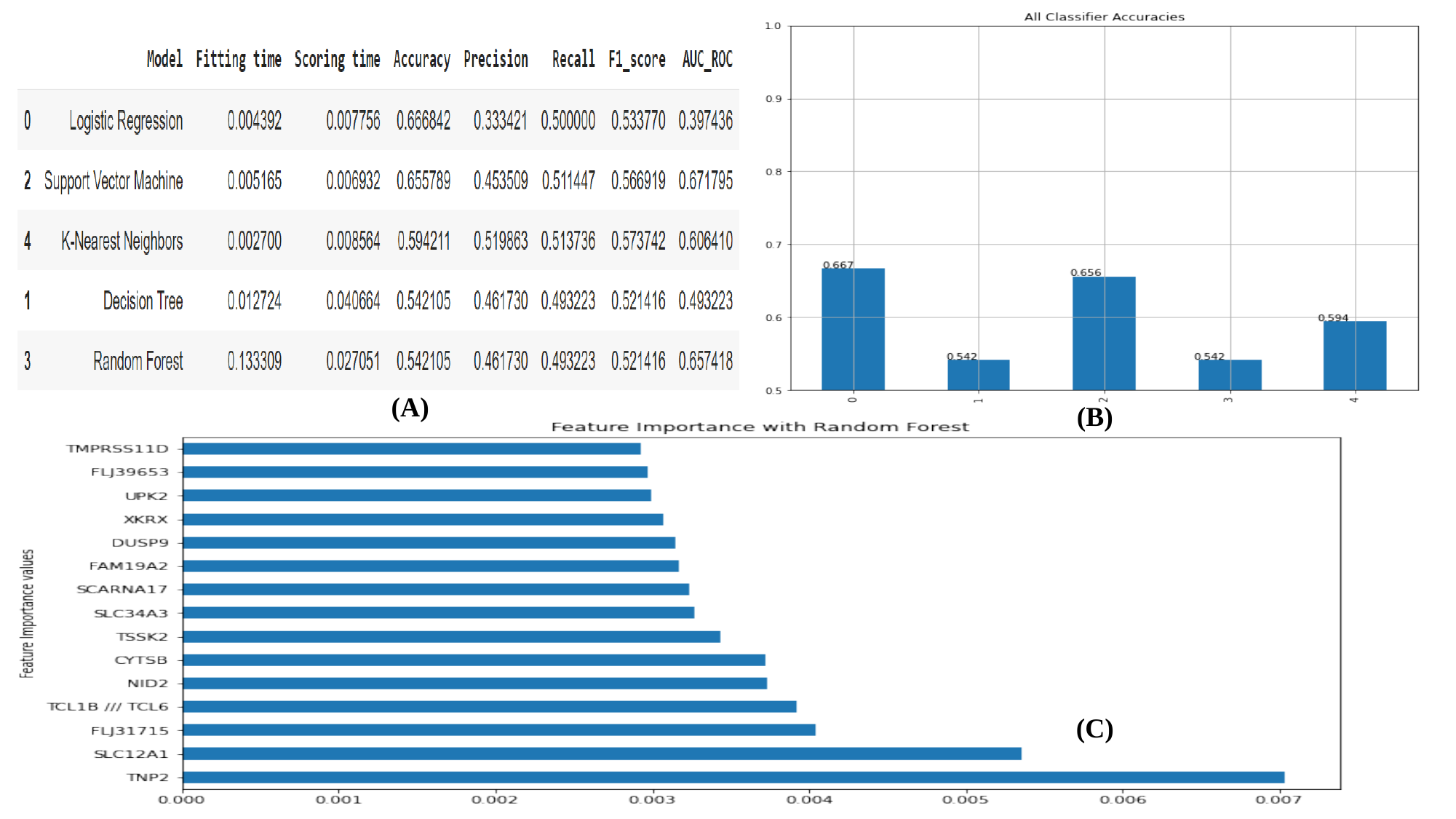

(A)
(B)
(C)

Supplement: Supplementary file 1 [file CG-24-84_SD1.zip › CG-24-84_SD1/Figure S7.pptx]

## Slide 1
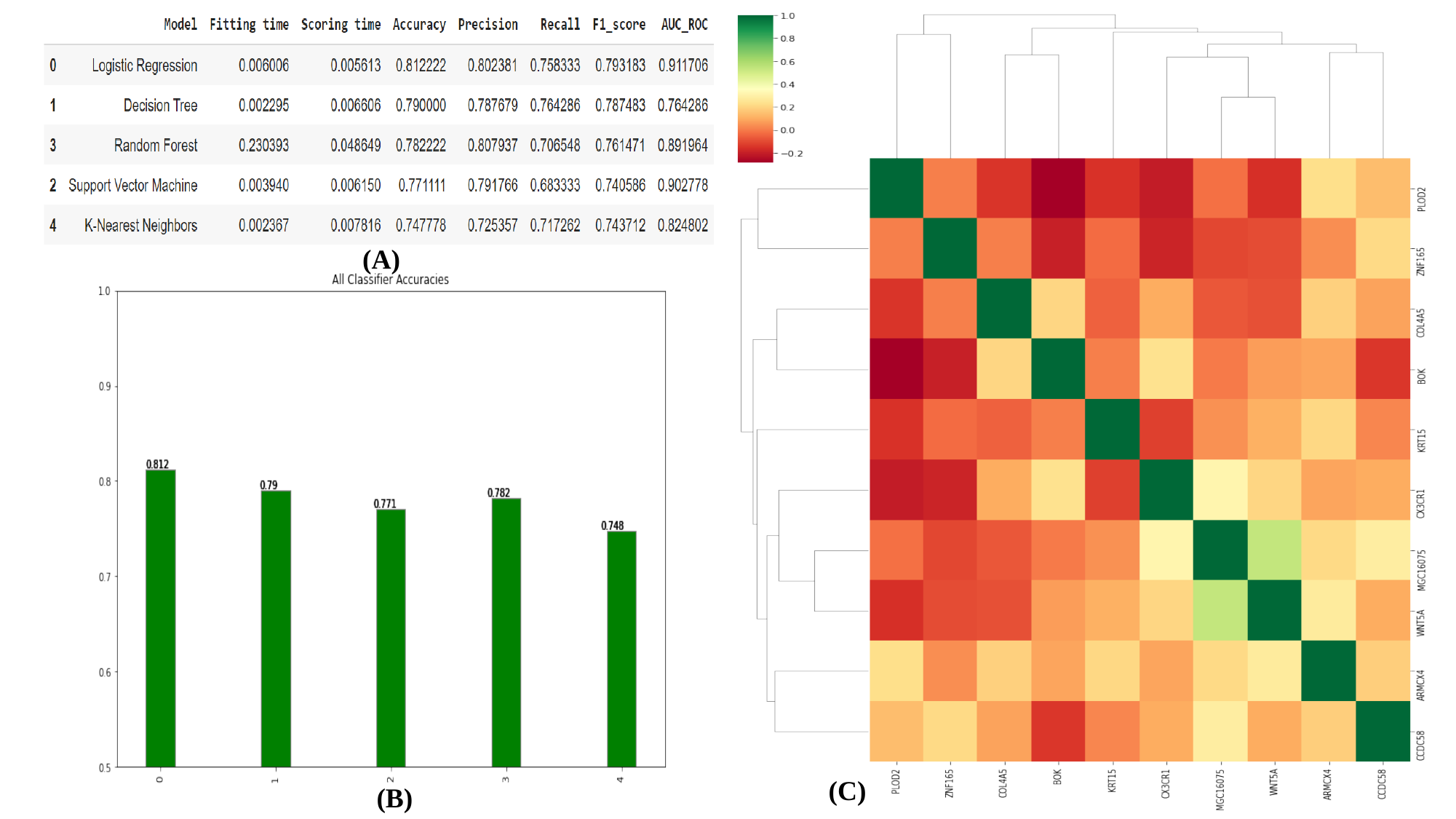

(A)
(C)
(B)

Supplement: Supplementary file 1 [file CG-24-84_SD1.zip › CG-24-84_SD1/Figure S8.pptx]

## Slide 1
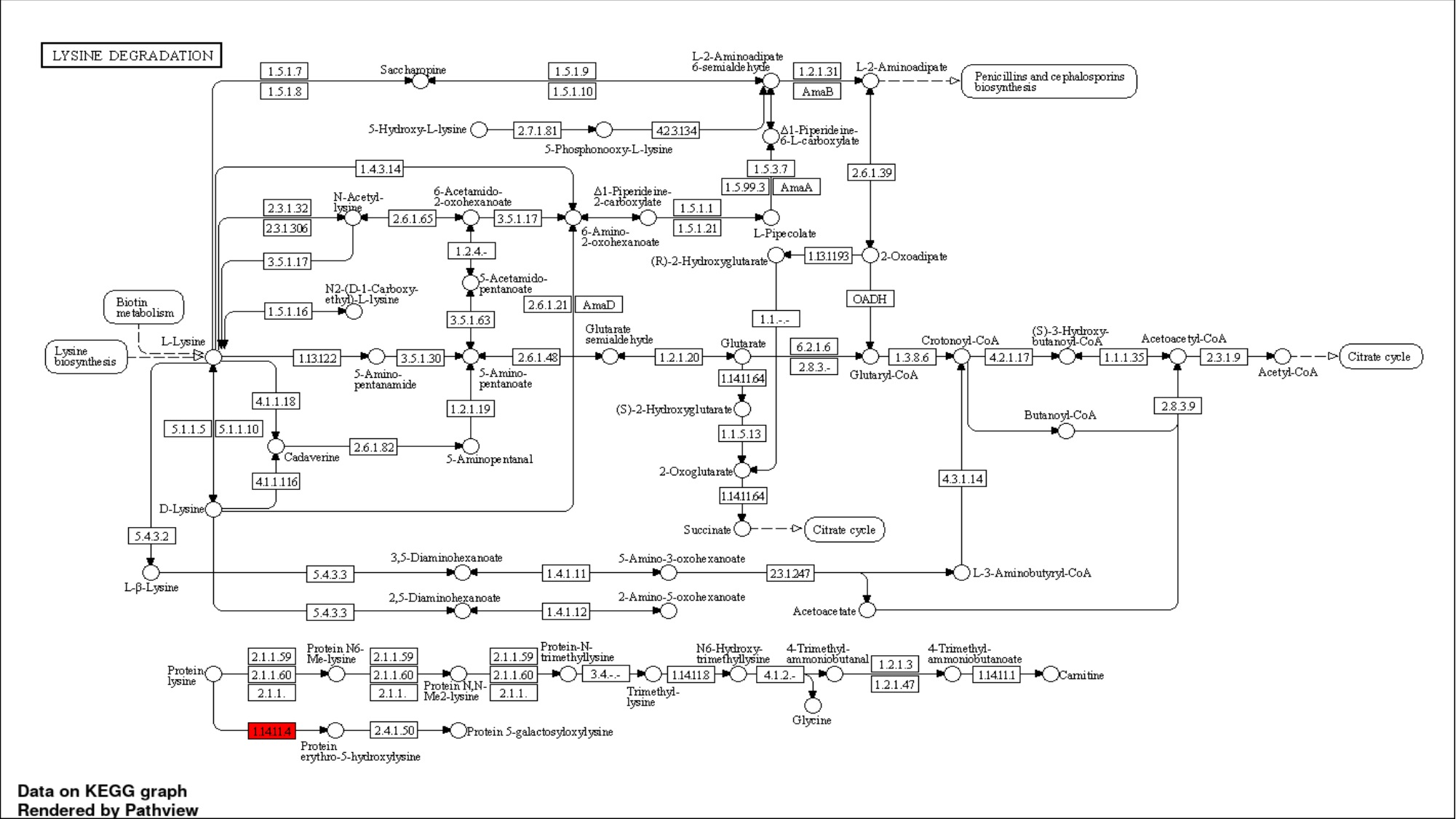

Supplement: Supplementary file 1 [file CG-24-84_SD1.zip › CG-24-84_SD1/Figure S9.pptx]
